# Shiga toxin targets the podocyte causing hemolytic uremic syndrome through endothelial complement activation

**DOI:** 10.1016/j.medj.2023.09.002

**Published:** 2023-10-19

**Authors:** Emily E. Bowen, Jennifer A. Hurcombe, Fern Barrington, Lindsay S. Keir, Louise K. Farmer, Matthew D. Wherlock, Carolina G. Ortiz-Sandoval, Valentina Bruno, Arlette Bohorquez-Hernandez, Daniel Diatlov, Niyousha Rostam-Shirazi, Sara Wells, Michelle Stewart, Lydia Teboul, Abigail C. Lay, Matthew J. Butler, Robert J.P. Pope, Eva M.S. Larkai, B. Paul Morgan, John Moppett, Simon C. Satchell, Gavin I. Welsh, Patrick D. Walker, Christoph Licht, Moin A. Saleem, Richard J.M. Coward

**Affiliations:** 1 Bristol Renal, Bristol Medical School, University of Bristol, Bristol BS1 3NY, UK; 2 The Hospital for Sick Children, Toronto, ON MG5 1x8, Canada; 3 MRC Harwell Institute, Mary Lyon Centre, Harwell Campus, Oxfordshire OX11 0RD, UK; 4 University of Mancheste, Manchester M13 9PT, UK; 5 UK Dementia Research Institute Cardiff and Systems Immunity Research Institute, School of Medicine, Cardiff University, Cardiff CF144XN. UK; 6 Bristol Royal Hospital for Sick Children, Bristol BS2 8BJ, UK; 7 Arkana Laboratories, Little Rock, AZ 72211, USA

## Abstract

**Background:**

Shiga toxin (Stx)-producing *Escherichia coli* hemolytic uremic syndrome (STEC-HUS) is the leading cause of acute kidney injury in children, with an associated mortality of up to 5%. The mechanisms underlying STEC-HUS and why the glomerular microvasculature is so susceptible to injury following systemic Stx infection are unclear.

**Methods:**

Transgenic mice were engineered to express the Stx receptor (Gb3) exclusively in their kidney podocytes (Pod-Gb3) and challenged with systemic Stx. Human glomerular cell models and kidney biopsies from patients with STEC-HUS were also studied.

**Findings:**

Stx-challenged Pod-Gb3 mice developed STEC-HUS. This was mediated by a reduction in podocyte vascular endothelial growth factor A (VEGF-A), which led to loss of glomerular endothelial cell (GEnC) glycocalyx, a reduction in GEnC inhibitory complement factor H binding, and local activation of the complement pathway. Early therapeutic inhibition of the terminal complement pathway with a C5 inhibitor rescued this podocyte-driven, Stx-induced HUS phenotype.

**Conclusions:**

This study potentially explains why systemic Stx exposure targets the glomerulus and supports the early use of terminal complement pathway inhibition in this devastating disease.

**Funding:**

This work was supported by the UK Medical Research Council (MRC) (grant nos. G0901987 and MR/K010492/1) and Kidney Research UK (grant nos. TF_007_20151127, RP42/2012, and SP/FSGS1/2013). The Mary Lyon Center is part of the MRC Harwell Institute and is funded by the MRC (A410).

## Graphical abstract

**Figure:**
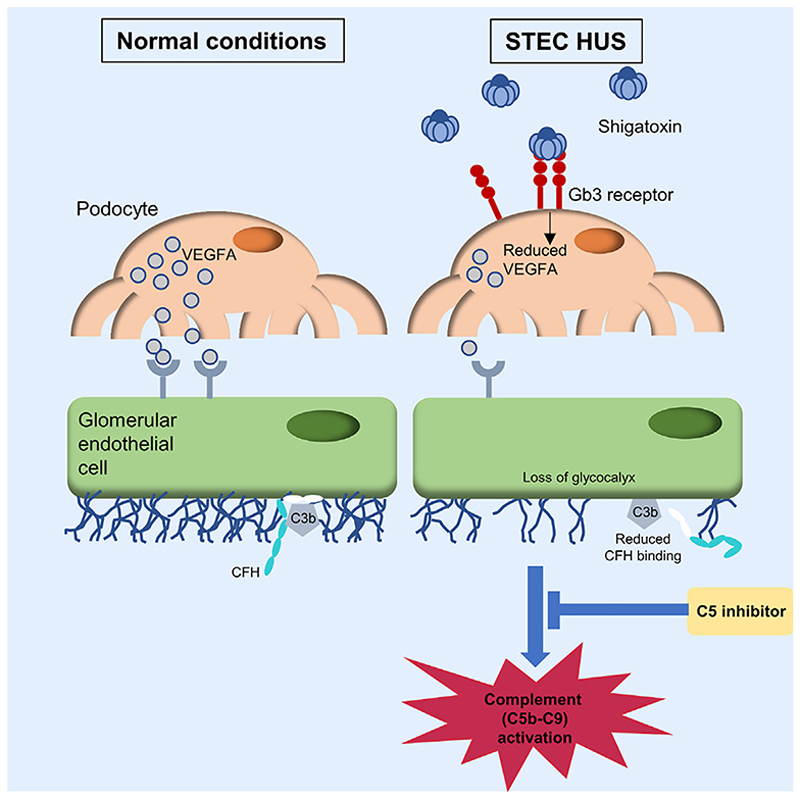
Bowen et al. explore the mechanisms underpinning the leading cause of kidney failure in children, Shiga-toxin-producing *Escherichia coli* hemolytic uremic syndrome (STEC-HUS). They discover that the podocyte is a key cell in this disease and that intravascular complement activation occurs early. Therapeutically blocking complement activation early prevents HUS developing.

## Introduction

Shiga toxin (Stx)-producing *Escherichia coli* hemolytic uremic syndrome (STEC-HUS) is the leading cause of dialysis-dependent acute kidney injury (AKI) in children and is associated with severe sequelae including end-stage renal disease (ESRD) or death in up to 5% of cases.^1^ In adults, a more severe clinical course is observed that results in ESRD or death in approximately 12% of cases.^2^ Clinically, it manifests as a triad of microangiopathic anemia, non-immune thrombocytopenia, and AKI.^1^ STEC-HUS is part of a group of systemic diseases termed “thrombotic microangiopathies” (TMAs), which are defined by common histopathological features with the potential to affect multiple organ beds including the kidneys, brain, gastrointestinal tract, respiratory system, and skin.^3^

Central to the development of TMAs is endothelial cell injury, leading to platelet activation and neutrophil recruitment and culminating in thrombus formation, inflammation, and end-organ damage.^4^ 90% of all HUS cases in children are caused by STEC from enteric ingestion of infected meat or soil, commonly resulting in hemorrhagic gastroenteritis, which allows the toxin to enter the circulation.^5^ However, exactly why the glomerular microvasculature is so susceptible to injury following systemic Stx infection remains unclear. Currently, there are no specific therapies for STEC-HUS, although there has been interest in the use of terminal complement inhibitors (such as eculizumab) in the treatment of the disease due to the benefit seen in atypical genetic forms of HUS^6,7^ where mutations in complement-regulating proteins promote unregulated amplification of the alternative pathway.

There are two main *E. coli* Stx variants, Stx1 and Stx2, of which Stx2 is more commonly associated with development of HUS in humans.^8^ The cellular receptor through which both Stx variants act is the neutral glycosphingolipid globotriaosyl-ceramide (Gb3/CD77).^9^ In humans, Gb3 is expressed in both glomerular endothelial cells (GEnCs) and podocytes.^5^ However, in mice, Gb3 expression is predominantly extra-glomerular,^10^ with Stx administration resulting in lethal renal tubular disease without histological evidence of glomerular TMA.^11^ Hence, there are no ideal mouse models of STEC-HUS that recapitulate human disease.^12^ Given this discrepancy in Gb3 expression across species, together with previous work from Eremina et al. showing that genetic knockdown of vascular endothelial growth factor A (VEGF-A) in the podocyte of adult mice caused a TMA,^13^ we hypothesized that podocyte Gb3 may be an important target receptor in STEC-HUS.

To explore our hypothesis, we engineered a transgenic mouse model in which Gb3 was exclusively expressed in the podocyte and gave these mice intraperitoneal Stx. Using human *in vitro* podocyte and GEnC co-culture models, immunohistochemical analysis of human kidney biopsies, and our novel mouse model, we show how podo-cyte-to-GEnC crosstalk contributes to GEnC injury in STEC-HUS.

## Results

### Generation of a podocyte-specific Gb3 mouse

It has already been established that Stx enters the cell via the neutral glycosphingolipid globotriaosylceramide (Gb3/CD77).^9^ Before engineering a mouse that exclusively expressed Gb3 in its podocytes, we first generated a Gb3 whole-body null mouse. This was achieved by globally deleting the Gb3 synthase enzyme (A4GALT), which catalyzes the addition of an α-1,4 galactose residue to lactosylceramide to form Gb3 (Figure S1). We confirmed Gb3 knockout using lipid mass spectrometry and endpoint PCR for Gb3 synthase from Gb3 null mice (Figures S1D, S1H, and S1I). Importantly, our Gb3 null mice had no overt phenotype and were completely protected from the toxicity of intraperitoneally injected Stx (Stx2), even at 2,000 times the lethal dose to 50% (LD_50_) of mice^14^ (Figures S1A and S1B) as previously reported.^15^ In contrast, all wild-type control mice died within 4 days of receiving Stx from extra-glomerular damage due to Gb3 expression in their renal tubules,^10^ as demonstrated by acute tubular necrosis on renal histology (Figure S1C).

To specifically study the role of the podocyte in the pathogenesis of STEC-HUS, we generated an inducible tetracycline-controlled (tet-on) podocyte-specific Gb3-expressing mouse on an otherwise Gb3 null background (Pod Gb3). This involved engineering a tet-on Gb3 synthase (A4GALT) mouse and crossing it with a podocin reverse tetracycline-controlled transactivator (rtTA) mouse to target the podocyte, with subsequent backcrossing with the Gb3 null mouse (Figures S1F and S1G). An inducible (rather than a constitutive Gb3-overexpressing) mouse model was chosen to avoid producing a podocyte Fabry’s disease phenotype caused by excessive accumulation of Gb3 in these cells.^16^

### Pod Gb3 mice develop HUS following Stx challenge

**Figure 1:**
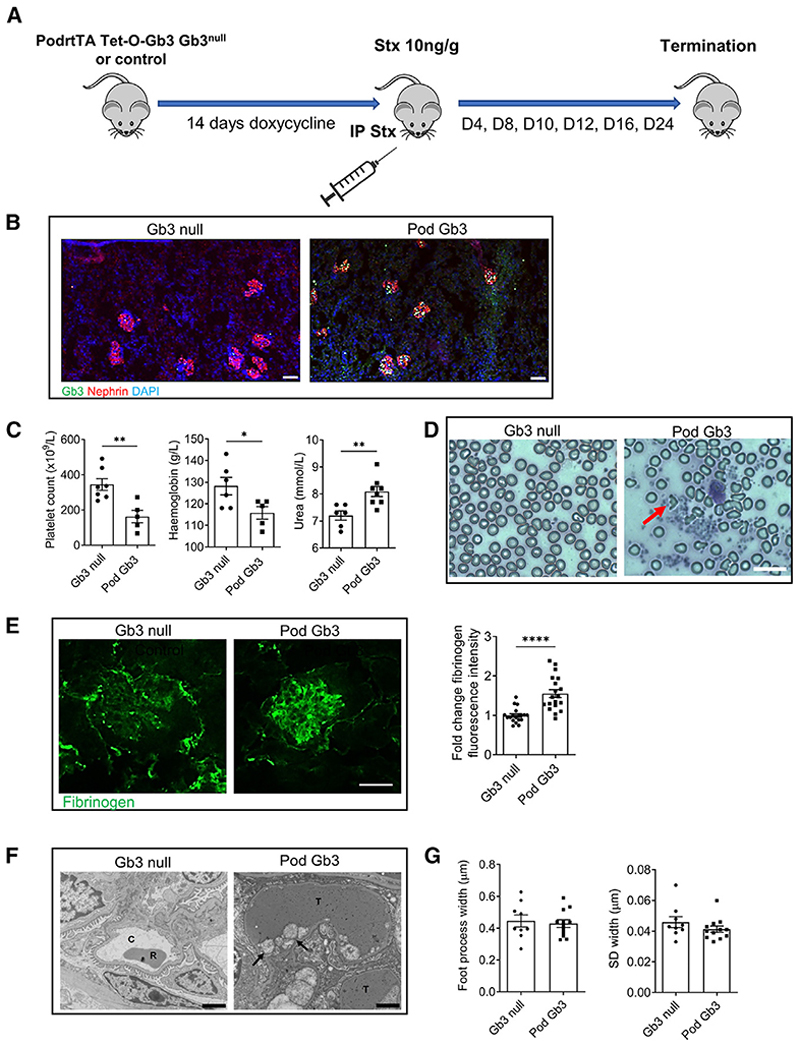
Shiga toxin causes HUS via the podocyte Gb3 receptor (A) PodrtTA-Tet-O-Gb3 Gb3 null (Pod Gb3 mice n = 28) and aged-matched PodrtTA-Tet-O-WT Gb3 null (control mice n = 25) were given 14 days of oral doxycycline to induce podocyte Gb3 expression, followed by 10 ng/g intraperitoneal (i.p.) Shiga toxin (Stx) inoculation. Mice were electively terminated at intervals of day 4, 8, 10, 12, 16, and 24 post-inoculations. (B) Immunofluorescence analysis for Gb3 expression in PodrtTA-Tet-O-WT Gb3 null (Gb3 null) control mice and PodrtTA-Tet-O-Gb3 Gb3 null (Pod Gb3) mice given 14 days of doxycycline to activate transcription of A4GALT (Gb3 synthase). Gb3 (green), nephrin (red), and DAPI nuclear stain (blue). Representative images for n = 3 mice for each genotype. Scale bar, 25 μm. (C) Blood samples were taken at the time of terminal anesthesia for platelet count, hemoglobin, and urea. Day 10 post-i.p. Stx shown. Unpaired t test p values: platelets **p < 0.01 (Pod Gb3 n = 5, GB3 null n = 7), hemoglobin *p < 0.05 (Pod Gb3 n = 5, GB3 null n = 6), and urea **p < 0.01 (Pod Gb3 n = 8, GB3 null n = 6). Data are expressed as mean ± SEM. (D) Representative blood films from Pod Gb3 (n = 7) and GB3 null control mice (n = 5). Red arrow indicates fragmented red cell. Scale bar 75 μm. (E) Day 10 post-i.p. Stx glomerular immunofluorescence analysis for fibrinogen (green) in Pod Gb3 mice and Gb3 null controls. Scale bar, 25 μm. Graph shows fold change in corrected total glomerular fluorescence (CTGF) intensity calculated using ImageJ analysis for fibrinogen deposition in the glomerulus. Pod Gb3 n = 3, Gb3 null n = 3 with 30 glomeruli per mouse analyzed. Unpaired t test p value: ****p < 0.0001. Data are expressed as mean ± SEM. (F) Transmission electron microscopy (TEM) from Pod Gb3 and GB3 null mice at day 10 post-i.p. Stx. T: thrombus in glomerular capillary loop; arrows indicate subendothelial accumulation of electrolucent flocculent material characteristic of TMA. C: capillary loop with red blood cell (R). Images representative of n = 3 for each genotype. Scale bar, 2 μm. (G) No statistically significant difference in podocyte foot process width or slit diaphragm (SD) width between the two groups. n = 3 for each genotype, with 3 glomeruli analyzed per mouse and 3 capillaries per glomeruli. Data are expressed as mean ± SEM.

Pod Gb3 mice were administered oral doxycycline (Dox) in their drinking water for 14 days to induce Gb3 synthase expression in their podocytes (Figure 1A). To confirm the expression of Gb3 in the podocytes of Pod Gb3 mice (PodrtTA-Tet-O-Gb3 Gb3 null) and the absence of expression in Gb3 null controls (PodrtTA-Tet-O-WT [wild type] Gb3 null), we performed endpoint PCR (Figure S1H) and immunofluorescence to confirm podocyte GB3 expression (Figures 1B and S1K).

Intraperitoneal Stx was then administered at a dose of 10 ng/g, and the mice were electively terminated at days 4, 8, 10, 12, 16, or 24. Examining all time points, we found that in Pod GB3 mice, day 10 was when urea levels were highest and platelet count at its lowest (before a subsequent reactive thrombocytosis detectable by day 24). Hemoglobin plateaued at day 12 before fully recovering (Figures S2B−S2D).

At 10 days, Pod Gb3 mice developed systemic features of HUS^11^ including a thrombocytopenia, microcytic hemolytic anemia (with red cell fragments observed on blood film microscopy), and uremia (Figures 1C and 1D). At day 10, Pod Gb3 mice also exhibited endotheliosis, glomerular fibrinogen deposition, and thrombi in their capillary lumens, which are the classical histological features of HUS (Figures 1E, 1F, and S2A). No structural alterations were detected in the podocytes at this time point using transmission electron microscopy (TEM) (Figure 1G).

### The glomerular intraluminal endothelial terminal complement cascade is activated in Pod Gb3 Stx mice and in patients with STEC-HUS

**Figure 2:**
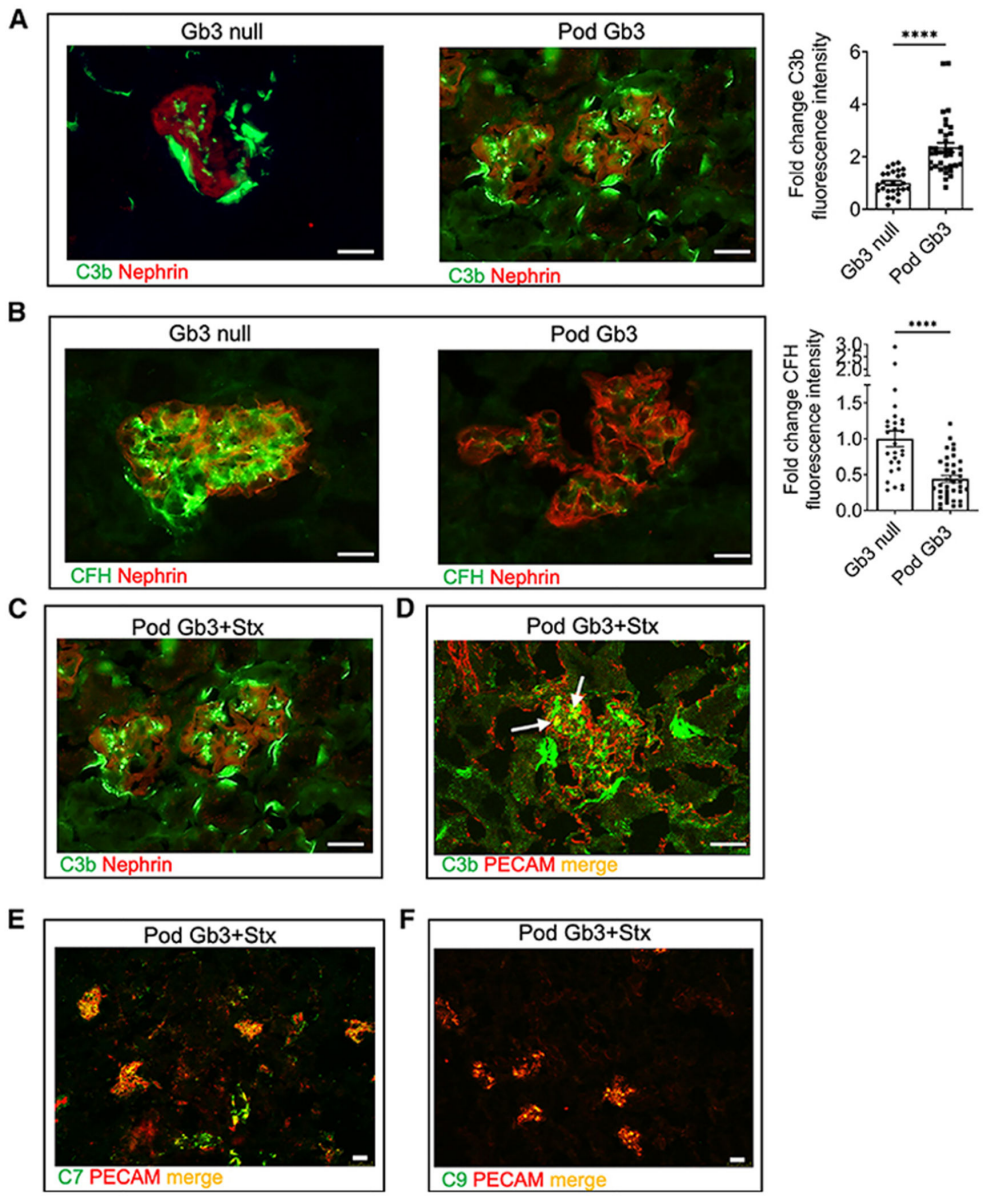
Stx binding to the podocyte Gb3 receptor causes complement deposition and a reduction in CFH binding on GEnC surfaces (A) Day 10 post-i.p. Stx glomerular immunofluorescence analysis for C3b (green) with co-staining for nephrin (red) in Pod Gb3 and GB3 null control mice glomeruli. Scale bar, 25 μm. Corresponding graph shows fold change in CTGF intensity, which was calculated using ImageJ analysis for C3b in the glomerulus. Pod Gb3 n = 3, Gb3 null n = 3 with 30 glomeruli per mouse analyzed. Unpaired t test p value: ****p < 0.0001.Data are expressed as mean ± SEM. (B) CFH (green) with co-staining for nephrin (red) in Pod Gb3 and GB3 null mice glomeruli. Scale bar, 25 μm. Corresponding graph shows fold change in CTGF intensity, which was calculated using ImageJ analysis for complement factor H deposition in the glomerulus. Pod Gb3 n = 3, Gb3 null n = 3 with 30 glomeruli per mouse analyzed. Unpaired t test p value: ****p < 0.0001.Data are expressed as mean ± SEM. (C) Glomerular immunofluorescence for C3b (green) co-localized with podocyte marker nephrin (red) Pod Gb3+Stx mice. Scale bar, 25 μm. (D) Glomerular immunofluorescence for C3b (green) with endothelial marker PECAM (red) in Pod Gb3+Stx mice. Scale bar, 25 μm. (E) Glomerular immunofluorescence for C7 (green) co-localized with endothelial marker PECAM (red) in Pod Gb3+Stx mice. Scale bar, 25 μm. (F) Glomerular immunofluorescence for C9 (green) co-localized with endothelial marker PECAM (red) in Pod Gb3+Stx mice. Scale bar, 25 μm.

Given recent interest in the use of complement inhibitors in the treatment of patients with STEC-HUS^17^ and reports that complement activation may contribute to the pathophysiology of STEC-HUS,^18,19^ we were interested to assess glomerular complement activation in our Pod Gb3 STEC-HUS mouse model. Using immunofluorescence on fresh frozen kidney sections we found an increase in C3b, C7, and C9 in Pod Gb3 mice 10 days after intraperitoneal Stx2 administration that was localized to the intraluminal glomerular endothelium using a PECAM co-stain. (Figure 2A, and 2C−2F). Furthermore, the inhibitory alternative complement pathway regulator, complement factor H (CFH) protein, was significantly reduced on the surface of the glomerular endothelium of the Stx2-treated Pod Gb3 mice, rendering them more susceptible to complement activation (Figure 2B). Examining our earlier time points, we found that terminal complement activation (C9) was present within 4 days of Stx2 challenge in the Pod GB3 mice (Figure S2E).

**Figure 3:**
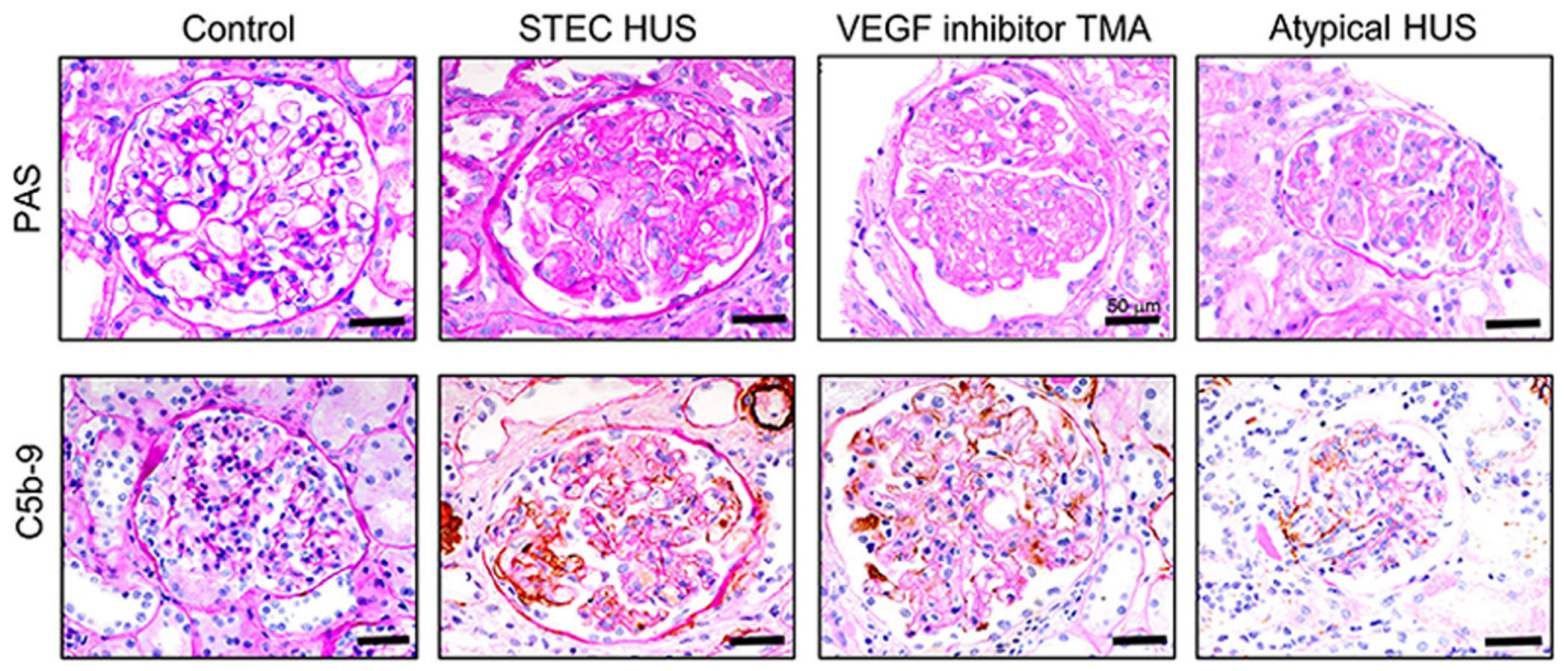
STEC-HUS leads to terminal complement activation in human kidney tissue Representative images of light microscopy and C5b-9 immunohistochemically stained human kidney sections from a healthy control patient, a patient with STEC-HUS, patient with a VEGF inhibitor, and a patient with atypical HUS. Light microscopy periodic acid Schiff (PAS) confirmed typical TMA features in the patients with STEC-HUS, a VEGF inhibitor, and atypical HUS with mesangiolysis, endocapillary swelling, and proliferation with GBM duplication (top). C5b-9 staining (brown) was negative in the control cases but positive in all others (bottom). Scale bar, 50 μm. See Figure S3 for all STEC-HUS cases (n = 3).

To assess whether terminal complement pathway activation occurs in the glomeruli of human patients with STEC-HUS, we examined a series of kidney biopsies from a variety of patients with proven STEC-HUS alongside appropriate control subjects for complement-driven glomerular TMA using C5b-9 immunohistochemical analysis (Table 1). We found that terminal complement activation (C5b-9) was present in the glomeruli of all patients with STEC-HUS studied (Figures 3 and S3).

### Podocyte Gb3-Stx binding *in vivo* reduces podocyte VEGF-A production

**Figure 4:**
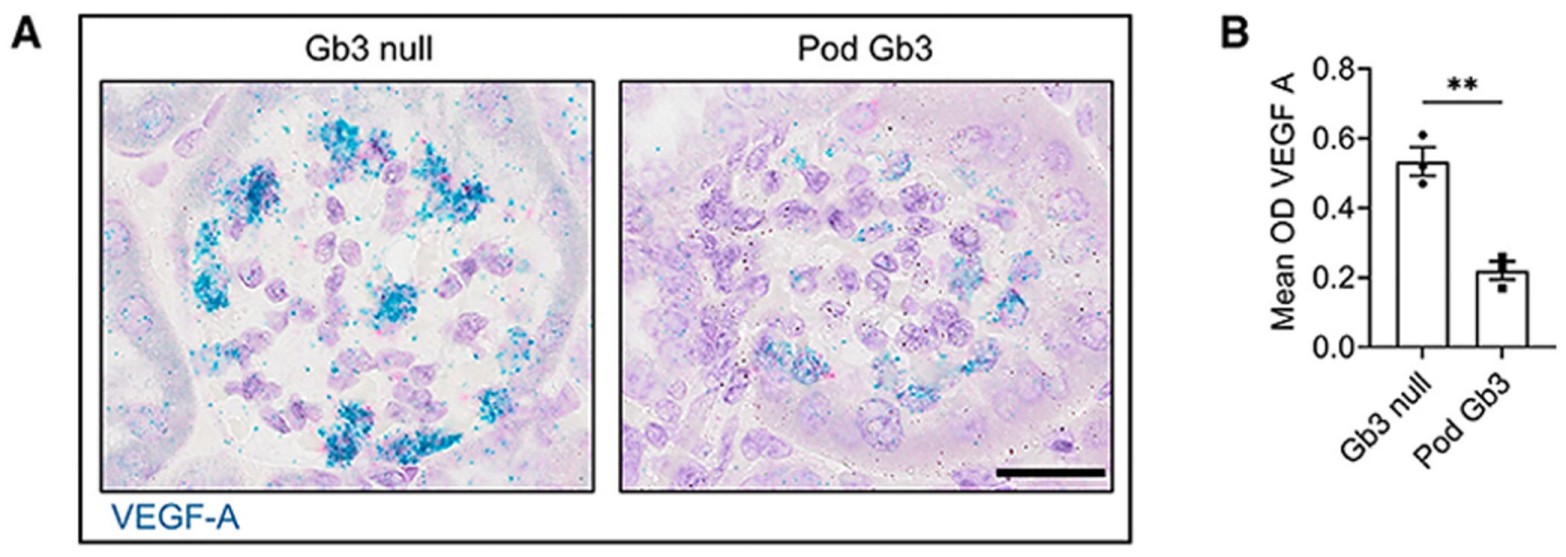
STEC-HUS results in a reduction in podocyte VEGF-A production (A) RNAscope *in situ* hybridization for VEGF-A mRNA in Pod Gb3 mice and GB3 null mice 10 days post-Stx inoculation. Representative images for n = 3 of each genotype. Scale bar, 50 μm. (B) Mean optical density (OD) for podocyte VEGF-A was calculated using the Quantitative Pathology and Bioimage Analysis program Qu Path in Pod Gb3 mice (n = 3) vs. controls (n = 3) at day 10 following i.p. Stx, with 15 glomeruli per mouse analyzed. Unpaired t test p value: **p < 0.01.Data are expressed as mean ± SEM.

In view of previous work by Eremina et al.,^13^ we were interested in ascertaining whether our Pod Gb3 Stx mouse model exhibited any alterations in VEGF-A expression. Using RNAscope *in situ* hybridization, we identified a significant reduction in podocyte VEGF-A expression in Pod Gb3 Stx mice compared with in GB3 null controls at day 10 (Figure 4). This suggests that Stx2 is affecting podocyte VEGF-A transcription, not just VEGF-A protein translation, as expected given its known ribotoxicity.^20^ Reduced podocyte VEGF-A production was also detectable by day 4, suggesting this is also an early event in the STEC-HUS process (Figure S4). We examined our human STEC biopsies for podocyte VEGF-A mRNA expression, but this was variable (in contrast to the C5-C9 activation) (data not shown). Unfortunately, for the human samples, and unlike the mice, we did not have optimal controls to analyze for changes in VEGF-A expression. Ideally, we would have examined human kidney biopsy specimens in STEC-HUS patients both during and after disease resolution to assess if their podocyte VEGF-A levels had increased, but this was not possible, as routine kidney biopsies are not performed.

### Human glomerular podocyte and GEnC co-culture mimics our *in vivo* findings

**Figure 5:**
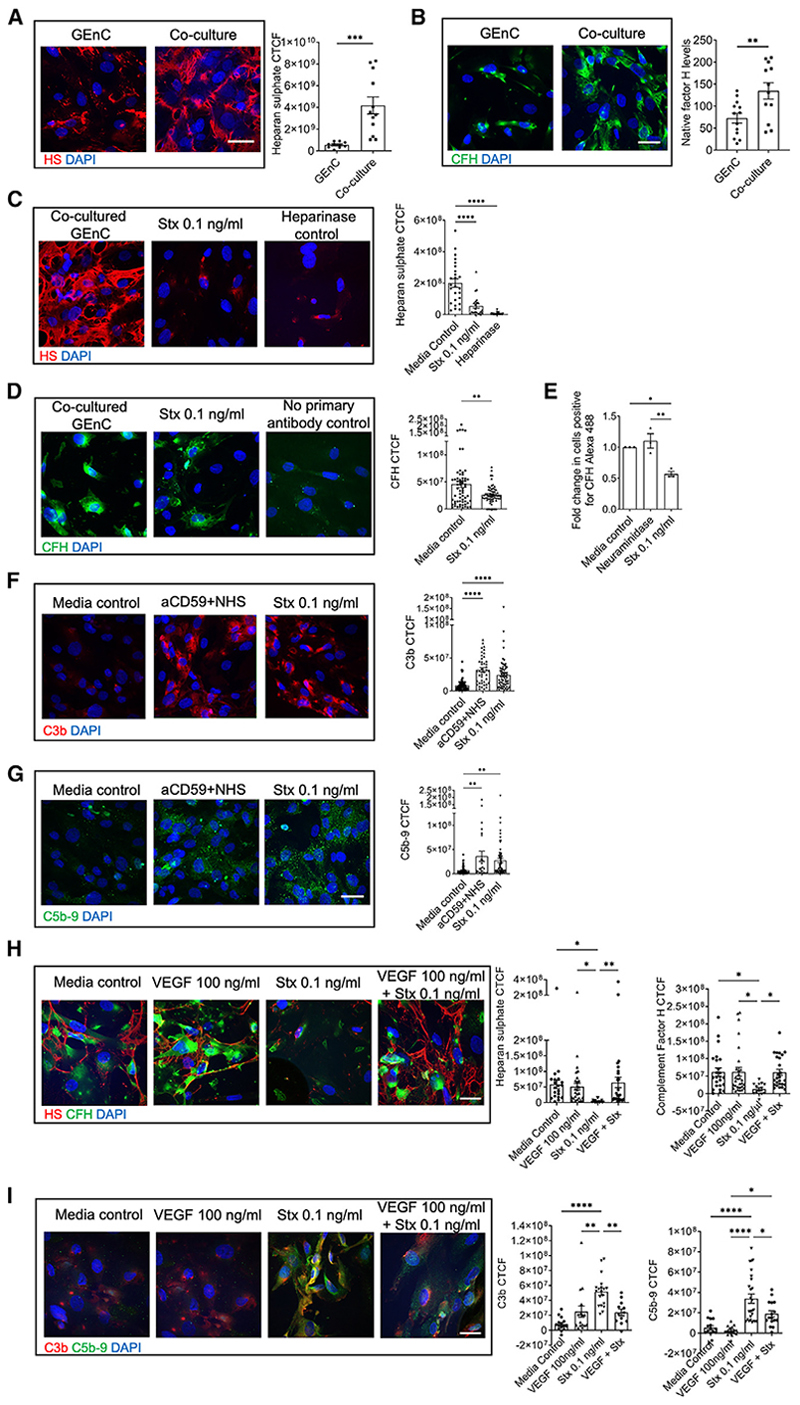
Human podocyte and GEnC co-culture experiments demonstrate glomerular cell crosstalk (A) Immunofluorescence images and corrected total cellular fluorescence (CTCF) intensity analysis of cell surface heparan sulfate (HS) expression in human conditionally immortalized GEnCs alone and co-cultured with podocytes. Scale bar, 50 μm. Unpaired t test p value: ***p < 0.001. Data are expressed as mean ± SEM. (B) Immunofluorescence images and CTCF analysis of complement factor H (CFH) expression in human conditionally immortalized GEnCs alone and co-cultured with podocytes. Scale bar, 50 μm. Unpaired t test p value: ***p < 0.01.Data are expressed as mean ± SEM. (C) Immunofluorescence images and CTCF analysis of cell surface HS expression in co-cultured GEnCs in media vs. co-cultured GEnCs where the podocyte transwell has been treated with 0.1 ng Stx for 15 min or co-cultured GEnCs directly treated with heparinase III 0.5 U/mL (as a heparan sulphate antibody negative control). DAPI staining in blue. Scale bar, 50 μm. One-way ANOVA with Tukey’s multiple comparison test p value ****p < 0.0001. Data are expressed as mean ± SEM. (D) Immunofluorescence images and CTCF analysis of CFH expression in co-cultured GEnCs in media vs. co-cultured GEnCs where the podocyte Transwell has been treated with 0.1 ng Stx for 15 min. DAPI staining in blue. Scale bar, 50 μm. Unpaired t test p value: **p < 0.01. Data are expressed as mean ± SEM. (E) Alexa Fluor 488-conjugated CFH binding on co-cultured GEnC surfaces was assessed by flow cytometry. Non-viable cells were excluded from analysis with Fixable Viability Dye eFluor 780. Data were normalized to media control and expressed as a fold change in mean fluorescence intensity following treatment of podocytes with neuraminidase (negative control) or 0.1 ng/mL Stx. n = 3 with each data point representing an n number. One-way ANOVA with Tukey’s post-test p values: *p < 0.05 and **p < 0.01.Data are expressed as mean ± SEM. (F) Immunofluorescence images and CTCF analysis of C3b (red) in co-cultured Transwell human GEnCs following podocyte treatment with either media (control), aCD59 and normal human serum (NHS) (positive control), or 0.1 ng/mL Stx. Scale bar, 50 μm. One-way ANOVA with Tukey’s multiple comparison test p value: ****p < 0.0001. Each data point on all graphs represents the average CTCF taken for each field of view, with at least 5 fields of view per condition and n = 4 experiments. Data are expressed as mean ± SEM. (G) Immunofluorescence images and CTCF analysis of C5b-9 (green) in co-cultured Transwell human GEnCs following podocyte treatment with either media (control), aCD59 and NHS (positive control), or 0.1 ng/mL Stx. Scale bar, 50 μm. One-way ANOVA with Tukey’s multiple comparison test p value: **p < 0.01. Each data point on all graphs represents the average CTCF taken for each field of view, with at least 5 fields of view per condition and n = 4 experiments. Data are expressed as mean ± SEM. (H) Immunofluorescence images of cell surface HS expression (red) and CFH (green) in co-cultured GEnCs treated with media (control), 100 ng/mL VEGF for 24 h, and 0.1 ng/mL Stx and 100 ng/mL VEGF (24 h pre-treatment) followed by 0.1 ng/mL Stx. Scale bar, 50 μm. CTFC analysis of cell surface HS and CFH levels also presented. One-way ANOVA with Tukey’s multiple comparison test p values: *p < 0.05 and **p < 0.01. Each data point on the graph represents the average CTCF taken for each field of view, with at least 5 fields of view per condition and n = 4 experiments. Data are expressed as mean ± SEM. (I) Immunofluorescence images of C3b (red) and C5b-9 (green) co-cultured GEnCs treated with media (control), 100 ng/mL VEGF for 24 h, and 0.1 ng/mL Stx and 100 ng/mL VEGF (24 h pretreatment) followed by 0.1 ng/mL Stx. Scale bar, 50 μm. CTFC analysis of C3b and C5b-9 expression also presented. One-way ANOVA with Tukey’s multiple comparison test p values: *p < 0.05, **p < 0.01, and ****p < 0.0001. Each data point on the graph represents the average CTCF taken for each field of view, with at least 5 fields of view per condition and n = 4 experiments. Data are expressed as mean ± SEM.

Following our *in vivo* observations in mice, that podocyte Stx2 signaling via the Gb3 receptor caused endothelial cell complement activation and a reduction in podocyte VEGF-A, we devised an *in vitro* human co-culture model to investigate the underlying molecular pathways involved in the crosstalk between human podocyte and GEnC. This utilized a 3 μm pore polyester membrane cell culture Trans-well insert, in which human conditionally immortalized podocytes were grown. Once terminally differentiated, they were then co-cultured with conditionally immortalized human GEnCs (Figure S5A). As we were interested in the role of VEGF-A in our model, GEnCs were cultured in endothelial cell media lacking additional VEGF-A. We found that GEnCs expressed significantly higher levels of both CFH and heparan sulfate (HS), a key component of the vascular endothelial glycocalyx,^21^ when co-cultured with podocytes in comparison to monoculture (Figures 5A and 5B).

We then used our co-culture model to examine the effect of podocyte Stx2 treatment on GEnC HS expression, CFH binding, and co-factor activity. To ascertain the effect of podocyte Stx2 injury on GEnCs, only the podocyte Transwell was treated with Stx2 at 0.1 ng/mL (Figure S5A). We performed permeability assays with a dextran-labeled 70 kDa tracer molecule (the same molecular weight as Stx2)^22^ to confirm that Stx2 could not permeate the Transwell 3 μm pores and therefore was not in direct contact with GEnCs (Figures S5B and S5C). Immunofluorescence analysis of GEnCs co-cultured with Stx2-treated podocytes showed a significant reduction in HS expression and CFH surface binding compared with untreated podocyte co-cultured GEnCs controls (Figures 5C and 5D). This was confirmed using flow cytometry techniques for CFH (Figure 5E). To establish whether this reduction in GEnC surface CFH led to complement activation, we assessed GEnCs cocultured with Stx2-treated podocytes for C3b and C5b-9 deposition using immunofluorescence. This was performed alongside media control co-cultured GEnCs (to ascertain baseline complement activity under co-culture conditions) and GEnCs treated with anti-CD59 and 50% normal human serum (NHS) as a positive control. We found that in GEnCs in normal culture conditions (with fetal calf serum [FCS] as a source of complement) co-cultured with Stx-treated podocytes, there was an increase in C3b and C5b-9 deposition on the endothelial cell surface compared with media-treated controls (Figures 5F and 5G). We then went on to examine if VEGF-A could rescue this phenotype. VEGF-A treatment of GEnCs protected them from podocyte Stx-induced loss of HS and reduced CFH binding (Figure 5H); it also prevented terminal complement activation (as shown by reduced C5-C9 activation) (Figure 5I). We also assessed the direct effects of Stx on human GEnC monoculture. Interestingly this also caused loss of glycocalyx, loss of CFH, and activation of complement. Impotantly, this process was also protected by VEGF-A (Figure S6), supporting the importance of glomerular crosstalk in the development of STEC-HUS.

### Terminal complement inhibition rescues the Pod Gb3 STEC-HUS phenotype

**Figure 6:**
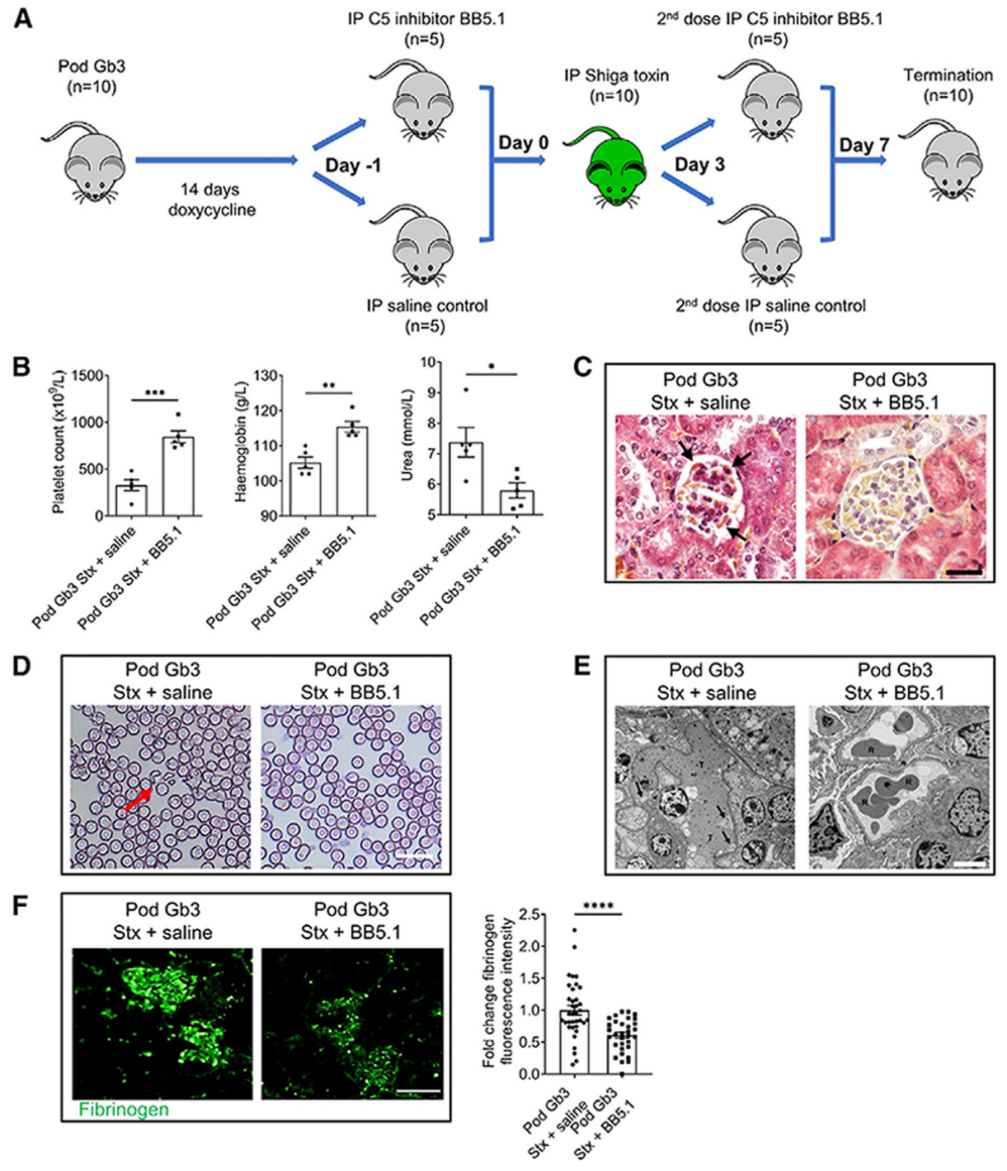
C5 inhibition rescues the Stx HUS phenotype (A) PodrtTA-Tet-O-Gb3 Gb3 null (Pod Gb3 mice n = 10) were given 14 days of oral doxycycline to induce podocyte Gb3 expression. Following randomization, they received either i.p. C5 inhibitor BB5.1 (n = 5) or sterile saline (n = 5). After 24 h, all mice received 10 ng/g i.p. Stx2 and, 72 h later, a further dose of i.p. C5 inhibitor or saline. At day 7 post-i.p. Stx, all mice were electively terminated. (B) Blood samples were taken at the time of terminal anesthesia for platelet count, hemoglobin, and urea (Pod Gb3+Stx+saline n = 5 and Pod Gb3+Stx+C5 inhibitor n = 5). Unpaired t test p values: platelets ***p < 0.001, hemoglobin **p < 0.01, and urea *p < 0.05. Data are expressed as mean ± SEM. (C) Light microscopy Martius Scarlet Blue (MSB) trichrome stain in Pod Gb3 mice following i.p. Stx and either saline or C5 inhibitor. Fibrin: red, collagen: blue, erythrocytes: yellow. Fibrin thrombi indicated by black arrows. Scale bar, 25 μm. (D) Representative blood films from Pod Gb3 post-i.p. Stx and either saline (control group n = 5) or C5 inhibitor (n = 5). Red arrow indicates fragmented red cell. Scale bar, 75 μm. (E) TEMs from Pod Gb3 post-i.p. Stx and either saline (control group n = 5) or C5 inhibitor (n = 5). T: thrombus in glomerular capillary loop; arrows indicate subendothelial accumulation of electrolucent flocculent material characteristic of TMA. R: red blood cell in capillary loop. Scale bar, 5 μm. (F) Glomerular immunofluorescence analysis for fibrinogen (green) in Pod Gb3 mice post-i.p. Stx and either saline or C5 inhibitor. Scale bar, 25 μm. Fold change in CTGF intensity was calculated using ImageJ analysis for fibrinogen deposition in the glomerulus. Pod Gb3+Stx+saline n = 4, Pod Gb3+Stx+C5 inhibitor n = 4 with 15 glomeruli per mouse analyzed. Unpaired t test p value: ****p < 0.0001. Data are expressed as mean ± SEM.

Given our observations both *in vivo* and *in vitro* that Stx2 leads to a reduction in GEnC CFH binding and subsequent endothelial complement activation, we attempted to rescue the HUS phenotype using the C5 complement inhibitor BB5.1. This is a murine immunoglobulin G1 (IgG1) antibody that blocks C5a activity and the formation of C5b-9 *in vivo*^23,24^ (analogous to eculizumab in humans) (Figure S7A). Pod Gb3 mice were given 14 days of Dox to induce podocyte Gb3 expression and randomized to receive either an intraperitoneal C5 inhibitor BB5.1 or saline control, 24 h before inoculation with 10 ng/g Stx2 (Figure 6A). Given that the half-life of BB5.1 is 72 h, a second dose was given (or saline to the control group) on day 3, supported by observations in other complement-driven murine disease models in which C5 has been successfully inhibited.^25^

C5 inhibition resulted in rescue of the STEC-HUS phenotype in the Stx-challenged Pod Gb3 mice. Thrombocytopenia, hemolytic anemia, and uremia were all prevented, with no histological evidence of glomerular TMA on Martius Scarlet Blue (MSB) stain or TEM (Figures 6B−6E). BB5.1-treated mice had reduced glomerular fibrinogen levels in comparison with saline-treated controls (Figure 6F). To confirm that therapeutic C5 blockade had been achieved, glomerular C7 and C9 deposition was assessed by immunofluorescence. This demonstrated terminal complement activation in saline-treated control mice, which was absent in Pod Gb3 BB5.1-treated mice (Figures S7B and S7C). As expected, C5 inhibition did not affect C3b glomerular deposition (Figure S7D). Together, these data support a role for complement inhibition in the treatment of patients with STEC-HUS.

## Discussion

In this study we have generated a rodent model that recapitulates STEC-HUS by targeting Stx to the glomerular podocyte by exclusively expressing the Gb3 receptor only in this cell type. Using this *in vivo* model, together with a human glomerular cell co-culture model, we demonstrate podocyte-to-GEnC crosstalk, whereby a rapid reduction in podocyte VEGF-A secretion leads to loss of GEnC glycocalyx, a reduction in GEnC CFH binding, and the resultant activation of the terminal complement pathway. Inhibiting terminal complement activation early in this model by targeting C5 prevented the development of STEC-HUS.

STEC-HUS is a devastating disease, caused by a systemically circulating toxin that has a predilection for the glomerulus of the kidney causing AKI. Our findings suggest that the reason the glomerulus is targeted in this disease is due to the action of Stx on podocytes, which are GEnC pericytes. Our initial hypothesis that podocytes were the target cell for STEC-HUS arose from the seminal work by Eremina et al. in which podocyte-specific knockout of VEGF-A in adult mice resulted in a glomerular TMA.^13^ Our work suggests that reduced podocyte VEGF-A is also a major driver in STEC-HUS. We have previously shown that, in both the glomerulus and the eye, a reduction in pericyte-produced VEGF-A signaling through the endothelial VEGFR2/PKC-α/CREB pathway is important in local complement activation via effects on complement regulatory CFH.^26^ Indeed, the critical importance of VEGF-A in protecting the endothelium from microangiopathic processes is further evidenced in human patients with cancer treated with VEGF inhibitors such as bevacizumab who develop glomerular TMAs.^13^ Collectively, these results suggest that VEGF-A protects the glomerular microvasculature not only through VEGFR2-mediated vasculotrophism but also through modulation of local CFH that could protect against complement-mediated damage. Thus, it appears that the podocyte VEGF-A axis is key in preventing TMA in STEC-HUS. Podocytes are the major source of VEGF-A in the kidney,^27^ and in our co-culture *in vitro* human models, VEGF-A protected the endothelium against Stx-mediated complement attack, restoring GEnC HS expression and factor H regulatory activity. It is interesting that other podocyte-driven glomerular diseases do not have a TMA picture. We speculate that this is due to the rapid loss of VEGF-A podocyte production in the mature glomerulus as this is common to both our model and also the previous work by Eremina et al.^13^ Understanding the dynamic regulation of podocyte VEGF-A production in differing kidney diseases should be pursued in more detail to understand how this key, podocyte-produced, growth factor^27,28^ regulates glomerular function.

Historically, mouse models of STEC-HUS analogous to human disease have been challenging to generate possibly due to the lack of glomerular expression of Gb3.^5,8^ Keepers et al. co-treated C57BL/6 mice with lipopolysaccharide (LPS) and Stx2 to augment Stx-mediated toxicity by proposed cytokine release and found that these mice developed AKI with features of HUS.^12^ However, this did not happen in isolation in these models, i.e., mice given Stx2 or LPS alone did not develop HUS. It is plausible that in this model, inflammatory cytokine production induced by LPS led to expression of glomerular Gb3, which would explain these findings. In our model, we found that expression of Gb3 was key in eliciting pathology, with Gb3 null mice completely protected from disease as previously reported by Okuda et al.^15^ Stx inoculation of WT mice led to severe acute tubular necrosis, reflecting renal tubular expression of Gb3 in the mouse (also reported by other groups^15,29^). However, our Pod Gb3 Stx-inoculated mice developed all the classical clinical features of HUS as well as glomerular TMA. Further understanding of the role of the Gb3 receptor elsewhere in the mouse in the context of Stx toxin exposure, particularly in organs where complications are seen (for example in the brain), will be of interest. Additionally, targeting Gb3 to the endothelium (in isolation and simultaneously with the podocyte) in the mouse should be performed to evaluate if this also causes a HUS picture in response to Stx challenge. Our model could easily be adapted to study this through the targeting of A4GALT (hence allowing GB3 expression) to other cell types by using different cellular rtTA drivers.

In recent years, the central role of alternative complement activation in rare inherited forms of genetic or atypical HUS (aHUS) has been established.^6^ Mutations in both complement inhibitory factors (including CFH, factor I, membrane co-factor protein [MCP / CD46], and thrombomodulin), proteins that promote alternative complement pathway amplification (C3 and factor B) and anti-factor H antibodies have been described.^6,7,30^ These inherited diseases have constant “low-grade” alternative complement activation and often present insidiously. When detected, they normally respond well to terminal complement inhibition via the C5 inhibitor eculizumab.^7^ Our studies support the notion that podocyte-to-GEnC crosstalk also activates the complement cascade early in the much more common form of HUS driven by Stx.

The role for complement in STEC-HUS has also been reported in other clinical studies. Children with STEC-HUS have been shown to have higher circulating levels of alternative complement pathway activation products including C3b, C3c, C3d, and factor B.^31,32^ Serological markers of alternative complement pathway activation and terminal complement complexes (C5b-9) have also been found in patients with STEC-HUS.^32,33^ Interestingly, studies have shown transient activation of the alternative and common complement pathway early in STEC-HUS.^19^ Unsurprisingly, following the success of C5 inhibition in genetic forms of HUS, there have been several case reports examining the potential benefit of eculizumab in STEC-HUS. These have been inconclusive; however, all studies have been in patients with established disease.^18^

Very recently, a phase 3 clinical trial from France (ClinicalTrials.gov: NCT02205541) has been reported.^34^ This trial recruited 100 children with STEC-HUS and randomized them to receive either eculizumab or a placebo. Its intention was to inhibit the terminal complement pathway early in STEC-HUS disease with inclusion criteria of an estimated glomerular filtration rate (eGFR) <75 mL/min/1.73 m^2^ (healthy being 120 mL/min/1.73 m^2^) and other features of STEC-HUS. Children with severe disease (including neurological, cardiac, or pancreatic involvement) were excluded. Unfortunately, early disease recruitment was not achieved, with most subjects having established renal disease before intervention as evidenced by the average eGFR of the children being 15.2 mL/min/1.73 m^2^ upon enrollment and 30% of them being dialysis dependent when the first dose of eculizumab/placebo was given. This important study showed no statistical difference in its primary endpoint relating to AKI, which was requiring less than 48 h of renal replacement therapy after first dose of eculizu-mab or placebo. It did show a significant improvement in renal parameters in the eculizumab-treated group at 1 year review (improved eGFR and/or blood pressure and/or proteinuria), but as the authors stated, this will need to be followed for several years to gauge its true significance. Importantly, there were no deaths from STEC-HUS disease or eculizumab in this trial, demonstrating no safety concerns of inhibiting the terminal complement pathway in this setting. Prophylactic penicillin and tetravalent meningococcal vaccinations were administered to all subjects as is standard practice.

Our work demonstrates that C5 inhibition can prevent Stx-driven HUS. But it should be noted that complement inhibition in our model was early, prior to giving Stx, which would not be possible clinically. However, it suggests that the early identification of STEC-HUS disease may be beneficial (e.g., through increased clinical awareness of the condition and/or the identification of early biomarkers), allowing prompt intervention with complement inhibitors to treat disease.

In conclusion, this study demonstrates the fundamental importance of the podocyte in the pathogenesis of STEC-HUS and provides the first *in vivo* evidence that an insult to the podocyte (in this case, Stx acting via the Gb3 receptor) can cause a glomerular endothelial phenotype in the form of TMA. Our findings also suggest that vascular bed specificity of systemic STEC-HUS to the glomerular endothelium is driven by podocytes acting as local pericytes and that complement activation is a major driver of pathology in this condition. This implies that early C5 inhibition has therapeutic potential in this devastating disease.

## Limitations of study

This work addressed the hypothesis that Stx can cause systemic HUS via the podocyte Gb3 receptor. Hence, we engineered a transgenic mouse model in which podocytes exclusively expressed Gb3. A more physiological model would have been to express GB3 both in the endothelium and podocytes before challenging with Stx. This is because Gb3 is expressed both in the podocyte and in the endothelium in humans. Our work shows that early inhibition of the terminal complement pathway in our model can rescue the HUS phenotype. We elected to inhibit this pathway prior to inducing disease with Stx. It would have been beneficial to have targeted C5 at different time points after inducing STEC-HUS disease and delineate the optimal time to inhibit complement activation. This is particularly important in view of the very recent publication from Garnier et al.^34^ However, using our model, we could also attempt to identify biomarkers in the blood and possibly the urine, which identify early disease activity, which could ultimately have clinical utility in the identification and subsequent early treatment of this disease. We intend to address these limitations in future work.

## Star+Methods

Detailed methods are provided in the online version of this paper and include the following:

- KEY RESOURCES TABLE
- RESOURCE AVAILABILITY
  ○ Lead contact
  ○ Materials availability
  ○ Data and code availability
- EXPERIMENTAL MODELS AND SUBJECT DETAILS
  ○ Mouse models
  ○ Cell lines
  ○ Transwell co-culture of podocytes and GEnCs
  ○ Podocyte permeability assay of transwell
  ○ Immunofluorescence staining of human cells
  ○ Flow cytometry factor H binding assay
  ○ Shiga toxin work
  ○ C5 inhibition
  ○ Endpoint PCR to detect Gb3 synthase
  ○ Urine and blood analysis
  ○ Histology and electron microscopy
  ○ Immunofluorescence staining of kidney sections
  ○ RNAscope in situ hybridisation
  ○ Immunofluorescence staining of human cells
  ○ Human samples
  ○ Immunohistochemistry for C5b-9 in human kidney tissue
  ○ Antibodies
- QUANTIFICATION AND STATISTICAL ANALYSIS

## Star+Methods

### Key Resources Table

**Table:** 

REAGENT or RESOURCE	SOURCE	IDENTIFIER
Antibodies		
Gb3/CD77	Beckman Coulter	#IM0175
Nephrin	2B Scientific	#BP5030
Podocin	Sigma Aldrich	#P0372
PECAM/CD31	Abcam	#ab124432
PECAM/CD31	BD Bioscience	#553370
C3b (mouse)	Hycult Biotech	#HM1065
C3d (Human) *non-specific for C3d also detects C3b and iC3b	Abcam	#ab17453
C3d (Human)	Quidel	#A207
Complement Factor H (mouse)	Hycult Biotech	#HM1119
Complement Factor H (human)	Quidel	#A312
Fibrinogen (FITC conjugated)	Dako	#F0111
C5b-9	Abcam	#ab66768
C7	Gift from Prof. Paul Morgan (Cardiff)	N/A
C9	Gift from Prof. Paul Morgan (Cardiff)	N/A
Anti-WT1 antibody	Merck	Clone 6F-H2 #05-753
Goat anti-Rabbit IgG (H + L) Cross-Adsorbed Secondary Antibody Alexa Fluor® 488	Thermo Fisher Scientific	#A11008; RRID:AB_143165
Goat anti-Guinea-pig IgG (H + L) Cross-Adsorbed Secondary Antibody Alexa Fluor® 568	Thermo Fisher Scientific	#A11075; RRID:AB_ 2534119
Anti-rabbit IgG peroxidase antibody	Sigma Aldrich	#A6667; RRID:AB_ 258307
Anti-mouse IgG peroxidase antibody	Sigma Aldrich	#A9044; RRID:AB_ 258431
Mouse anti-Heparan Sulfate (10E4 epitope)	US biological	#H1890
Bacterial and virus strains		
Shiga Toxin (Stx2)	List biological labs Inc	#162 lot 1621A1, Shiga toxin 2 from E.coli
Biological samples		
Patient kidney biopsy tissue	Arkana Laboratories, Little Rock, Arizona, USA	REC Protocol#2022/04/9, IRB00008523.
Chemicals, peptides, and recombinant proteins		
Human VEGF-A	Lonza	#CC-3125
Heparinase III	Sigma	#H8891-5U
Neuraminidase	New England Biolabs	#P0720L
Human Factor H	CSL Behring	N/A
Human Factor I	EMD Millipore Corp	#341280
Human Factor 3b	EMD Millipore Corp	#204860
Bovine serum albumin	Merck	#A9647
Hematoxylin Solution, Gill No. 1	Sigma	#GHS132
EGM-2 MV Microvascular EC Growth Media	Lonza	#CC-3202
RPMI 1640	Sigma	#R8758
C5 inhibitor (BB5.1)	Hycult® Biotech	#HM1073
Critical commercial assays		
Trichrome Staining Kit (MSB stain)	Atom Scientific Ltd.	#RRSK2-100
RNAScope wash buffer	Biotechne	#310091
RNAScope target retrieval reagents	Biotechne	#322000
RNAScope protease plus	Biotechne	#322330
RNAScope 2.5 HD duplex detection reagents	Biotechne	#322500
RNAScope probe Mm VEGF-A	Biotechne	#405131
RNAScope probe Mm WT1-C2	Biotechne	#432711-C2
RNAScope probe Hs VEGF-A	Biotechne	#423161
RNAScope probe Hs WT1-C2	Biotechne	#415581-C2
Corning 12mm Transwell 3um pore insert plates	Sigma	#CLS3402
Periodic Acid Schiff staining kit	Sigma	#395B
DNeasy Blood and tissue kit	Qiagen	#69504
SignalStain Boost IHC detection reagent (HRP rabbit)	Cell Signaling	Cat# 8114
SignalStain DAB substrate kit	Cell Signaling	Cat# 8059
Experimental models: Cell lines		
Conditionally immortalised human podocytes	Made in house (Keir et al.).	N/A
Conditionally immortalised human glomerular endothelial cells	Made in house (Satchell et al.).	N/A
Experimental models: Organisms/strains		
A4GALT KO mice (C57BL/6N-A4galt ^tm1b(EUCOMM)Hmgu^/H)	MRC Harwell	This paper
PodrtTA-Tet-O-Gb3 mice	MRC Harwell	This paper
Pod-rtTA; Tg(TetO-Gb3); A4galt ^tm1b/tm1b^	MRC Harwell	This paper
Pod-rtTA, A4galt ^tm1b/tm1b^	MRC Harwell	This paper
Oligonucleotides		
A4GALT Forward 5' TCTTCTTCCTAGAGACATCGGAC 3'	Eurofins Genomics	This paper
A4GALT Reverse 5' CCCTTTCATCAGCACAACCA 3'	Eurofins Genomics	This paper
Software and algorithms		
ImageJ	NIH	https://imagej.nih.gov/ij/download.html RRID:SCR_003070
Leica Application Suite X software	Leica Microsystems	https://www.leica-microsystems.com/products/microscope-software/details/product/leica-las-x-ls/; RRID:SCR_013673
GraphPad Prism	GraphPad Software, San Diego, CA, USA	Version 9.4.0 RRID:SCR_002798
Other		
Fetal bovine serum	Sigma	#F7524
Vectamount	Vector laboratories	#H-500
DPX mount for histology	Sigma	#06522

## Resource Availability

### Lead contact

Further information and requests for resources and reagents should be directed to and will be fulfilled by the lead contact, Professor Richard Coward (Richard.Coward@bristol.ac.uk).

### Materials availability

Mouse lines (A4GALT KO (C57BL/6N-A4galt ^tm1b(EUCOMM)Hmgu^/H)) and (Pod-rtTA; Tg(TetO-Gb3); A4galt ^tm1b/tm1b^ and Pod-rtTA, A4galt ^tm1b/tm1b^ controls) generated in this study have been deposited to MRC Harwell via the European Conditional Mouse Mutatgenesis (EUCOMM) and the National Institutes of Health Knockout Mouse (KOMP) programs embryonic stem (ES) cell repository.

## Experimental Models and Subject Details

### Mouse models

A4GALT KO mice (C57BL/6N-A4galt ^tm1b(EUCOMM)Hmgu^/H) were generated through a collaboration with MRC Harwell; through use of the International Mouse Phenotyping Consortium (IMPC) via the European Conditional Mouse Mutatgenesis (EUCOMM) and the National Institutes of Health Knockout Mouse (KOMP) programs embryonic stem (ES) cell resource. This repository was used to obtain mice containing the full Tm1a knock-out-first reporter tagged insertion allele upstream of a critical exon within the A4GALT gene (A4galt ^tm1a(EUCOMM)Hmgu^). This mouse was then crossed with mice expressing Cre recombinase (C57BL/6NTac-Tg(ACTB-cre)^3Mrt^/H). The progeny from this breeding was then genotyped by MRC Harwell using a combination of qPCR assays to identify the alleles generated. Only mice con-taining Tm1b converted forms of the original Tm1a allele were taken forward for use in experiments after a further backcross to remove the cre recombinase.

PodrtTA-Tet-*O*-Gb3 mice were generated by MRC Harwell by crossing the podocin rtTA (reverse tetracycline activated transactivator) mouse with the Tet-*O*-Gb3 synthase mouse (Tg(TetO-Gb3)). The Tet-*O*-Gb3 synthase construct (Extended Data Figure 1E) was devised in Bristol and sent to MRC Harwell, where it was re-transformed, linearised and injected into the nucleus of a fertilised C57BL/6N mouse oocyte. Founders from this pro-nuclear injection process were genotyped by MRC Harwell. To generate a mouse that only expressed Gb3 in the podocyte, Tg(TetO-Gb3) mice were crossed to A4galt ^tm1b/+^ and separately, Pod-rtTA mice were crossed to A4galt ^tm1b/+^. The two double heterozygous lines were then crossed to A4galt ^tm1b/tm1b^ to generate mice that were Tg(TetO-Gb3); A4galttm1b/tm1b; and mice that were Pod-rtTA A4galt ^tm1b/tm1b^. These mice were then inter-crossed to generate Pod-rtTA; Tg(TetO-Gb3); A4galt ^tm1b/tm1b^; and Pod-rtTA, A4galt ^tm1b/tm1b^ controls.

To induce podocyte Gb3 expression, 6−8 week old PodrtTA-Tet-*O*-Gb3 Gb3 null mice along with PodrtTA-Tet-*O*-WT Gb3 null littermate controls were given doxycycline (Sigma, Cat# D9891) via their drinking water (2 mg/mL doxycycline in 5% sucrose) for 14 days.

Tg(TetO-Gb3) and A4galt ^tm1b^ mice were co-isogenic on C57BL/6N, Pod-rtTA were on a mixed genetic background containing FVB/N, 129SV and DBA/2J. Therefore, the experimental cohort was on a mixed genetic background. Both sexes were initially studied in the Stx2 challenge experiments with no phenotypic differences observed. For our C5 inhibition experiments female mice only were studied.

Transgenic mouse work was carried out in accordance with the University of Bristol’s institutional guidelines and procedures approved by the United Kingdom (UK) Home Office in accordance with UK legislation (Home Office Protocol numbers PPL 3003394 & PP2012285).

### Cell lines

Conditionally immortalised podocyte cell lines^35^ were cultured in RPMI 1640 (Sigma, Cat# R8758) supplemented with 10% Fetal Bovine Serum (FBS) (Sigma, Cat# F7524) and 1% penicillin/streptomycin (Merck, Cat# P4333). Cells were initially grown at 33°C to allow proliferation before switching to 37°C for 10−14 days to allow cells to differentiate. Cell lines were authenticated by western blotting for typical podocyte marker proteins. Conditionally immortalised human glomerular endothelial cells (GEnCs)^36^ were grown at 33°C in humidified incubators in the presence of 5% CO_2_ in glomerular endothelial cell media: EGM-2MV with 5% FBS and additives (hEGF, hydrocortisone, hFGF-B, ascorbic acid and gentamicin, but in the absence of VEGF) (Lonza, Cat# CC-3202). They were grown until 70−80% confluent and thermo-switched to 37°C to differentiate for 5−7 days. All cells were used at a passage less than 25.

### Transwell co-culture of podocytes and GEnCs

Conditionally immortalised human podocytes were cultured under standard conditions as above and seeded in Corning 12mm transwell 3um pore insert plates (Sigma, Cat# CLS3402). Once established at 70−80% confluency they were thermo-switched to 37°C to differentiate for 10−12 days. After 3 days GEnCs were plated in 12 well plates and allowed to reach a confluency of 70−80% before being co-cultured with the aforementioned podocytes at 37°C. This allowed the glomerular cells (both podocytes and GEnCs) to be co-cultured for 5−7 days. Treatments were then applied to the podocyte transwell or GEnC well as required for each experiment.

### Podocyte permeability assay of transwell

Permeability assay experiments were performed to assess whether Stx could enter the GEnC compartment of the 12 well plate below. Podocytes were seeded into the transwell compartment of the co-culture plate and allowed to proliferate at 33°C until the bottom of the transwell was 70−80% confluent at which point they were thermo-switched to 37°C to terminally differentiate as before. After 10−12 days at 37°C a tracer molecule: 70kDa dextran, (ThermoFisher Scientific, Cat# D1823) which is the same molecular weight as Stx +/− Stx, +/− inomycin (Sigma, Cat# I3909) used as a way of injuring podocytes to increase their permeability and act as a positive control) were added to the transwell. A transwell with no cells was used as a positive control for tracer molecule detection. After 15 min incubation the plate was read on a bioluminescent plate reader and the light emitted from each well measured.

### Immunofluorescence staining of human cells

Cells were grown in 6 or 12 well plates with glass coverslips placed in each well. Once fully differentiated cell treatments were applied directly or to the transwell compartment above. Once the assay was completed, cells were washed 3 times in phosphate buffered saline (PBS) and fixed with ice-cold 4% PFA (paraformaldehyde). After 10−15 min PFA was washed off with PBS and permeabilised with 0.3% Triton X-100 for 10 min. All cells were blocked in 3% bovine serum albumin (BSA, Merck, Cat# A9647) for 60 min. Primary antibodies were then applied to the coverslips which were left at 4°C overnight. The following day, cells were washed 3 times in PBS and secondary fluorescent antibodies applied for 1 h at room temperature. Coverslips were then washed again 3 times and mounted with VECTA Shield hard setting mount with DAPI nuclear stain (Vector Lab Inc Cat# H100). Slides were then imaged using the Leica DMI 6000B microscope or Leica Confocal SP5-II confocal laser scanning microscope at the Wolfson Imaging Institute, University of Bristol; or with the Quorum Spinning Disk 2 confocal laser microscope at the SickKids Imaging Facility, Toronto, Canada. Image analysis was performed with ImageJ (RRID:SCR_003070); all images were contrast enhanced using the same parameters.

### Flow cytometry factor H binding assay

Binding of complement factor H to GEnC surfaces was demonstrated by flow cytometry as previously described, using purified CFH (CSL Behring, Marburg, Germany) tagged with Alexa Fluor 488 succinimidyl ester (10 μg/mL, Life Technologies, Cat# A20000) for 1 h at room temperature before being dialyzed overnight in PBS.^37^ Alexa Fluor 488-conjugated factor H was added for 3 min to live GEnCs before analysis with flow cytometry. GEnCs were either cultured alone (monocultured) or with podocytes (co-cultured). For each experimental run (each n) monocultured GEnCs and co-cultured GEnCs were in parallel (i.e., plated, thermo-switched, treated and analyzed at the same time) to control for any variability in physiochemical culture environment and allow true comparison between the two conditions.^38^ Monocultured GEnCs were treated with media lacking VEGF-A (baseline control), neuraminidase 500U/ml (which cleaves sialic acid groups from glycoproteins and is therefore a positive control for glycocalyx stripping^39^), hyaluronidase 20U/ml (another positive control for glycocalyx breakdown^40^) or Stx2 0.1 ng/ml. Co-cultured GEnCs were also grown in media lacking VEGF-A and only the podocyte transwell treated with either media lacking VEGF-A (baseline control), neuraminidase 500 U/ml or Stx2 0.1 ng/ml. All incubations were performed and removed prior to live GEnC flow cytometry analysis with Alexa Fluor labeled Factor H.

### Shiga toxin work

Shiga toxin 2 (Stx2) was obtained commercially from the USA through List Biological Laboratories Inc. (product #162 lot 1621A1). Stx2 was chosen as it is known to be more cytotoxic in mice than Stx1; and because epidemiological data from human studies have demonstrated that Stx2 producing STEC have a stronger association with more severe disease.^22^ All work involving the toxin was performed in a biological cabinet under category 3 facilities. Prior to any toxin work commencing, a ‘control of substances hazardous to health regulations’ (COSHH) risk assessment was completed which was submitted and reviewed by the biological and genetic safety committee.

Upon receipt of Shiga toxin, the lyophilized powder was reconstituted with 100μL distilled water and gently mixed by inversion in-keeping with the manufacturer’s instructions. This resulted in a stock concentration of 10μg/100μls which was aliquoted into 20μL stock eppendorfs and stored at −20°C until use. The toxin was assessed for purity on 12% SDS-PAGE and was found to have two major bands at molecular weights of 33 kDa and 8 kDa corresponding to A and B toxin subunits, respectively. Purity was >98% and the endotoxin content determined on kinetic chromogenic LAL assay at List Labs was <90EU/mg. A dose of 10 ng/g of Shiga toxin diluted in sterile normal saline 0.9% (Baxter) was given to each mouse via intraperitoneal injection. All *in vitro* Shiga toxin cell treatments were performed on differentiated cells at a dose of 0.1 ng/ml.

### C5 inhibition

PodrtTA-Tet-*O*-Gb3 Gb3 null mice aged between 8 and 10 weeks were given 14 days of oral doxycycline (Sigma, Cat# D9891) in their drinking water to induce Gb3 expression in their podocytes. They were then randomised to receive either 1mg of intraperitoneal (IP) mouse C5 inhibitor (mAb BB5.1 Hycult Biotech, #HM1073) or IP 0.9% sterile normal saline of the same volume as a control. The following day all mice received IP Shiga toxin at a dose of 10 ng/g. Three days later the mice were given either a second dose of 1mg of IP C5 inhibitor or IP 0.9% sterile normal saline control (Figure 6A). This dosing regime was chosen due to the half-life of BB5.1 which is 72 h, together with the recommendation of Hycult Biotech of twice weekly dosing which has been reported by other groups to successfully inhibit C5 *in vivo*.^23,25^

### Endpoint PCR to detect Gb3 synthase

Genomic DNA was isolated from mouse tissues using a blood and tissue DNA extraction kit (DNeasy Blood and tissue kit, Qiagen, Cat# 69504). 500−1000 ng DNA was used in endpoint PCR reactions using hotmaster taq polymerase (5 Prime). Primers (obtained from Eurofins Scientific) were used at a concentration of 10μM and 1μL of cDNA sample used as standard for each reaction. The Tm was set according to Eurofins data sheet which was provided with each primer pair. Endpoint PCR was used to identify murine A4GALT expression using the following primer pair: Forward 5′ TCTTCTTCCTAGAGACATCGGAC 3′ and Reverse 5′ CCCTTTCATCAGCAC AACCA 3’. The amplification product (after PCR clean up using Qiagen PCR purification kit) was sent for sequencing to Eurofins Genomics in Germany to confirm the specificity of the primers.

### Urine and blood analysis

Urine Albumin:creatinine ratio (ACR) quantitative analysis was performed using a mouse-specific albumin ELISA (Universal Biologicals, Cat# E90-134) and creatinine companion kit (Exocell, Cat# 1012), following the manufacturers methodology. Blood samples were taken from mice at the time of terminal anesthesia via cannulation of the inferior vena cava with a 23 gauge needle and pre-heparinised syringe (30−50 USP of heparin (Sigma, Cat# H6279) per mL of blood collected was used as per convention).^41^

Blood films were produced by hand, taking a small drop (<5 μL) of blood on a Claritex glass histology slide using a wooden stick. Another Claritex slide was then placed on top of the first, the short edge held at an angle of 30° and the blood droplet allowed to spread across the short edge (by capillary action). The top glass slide was then swept across the bottom slide to spread the blood across it in one smooth motion; leaving a trail of blood that goes from a thicker smear through to a thinner film of blood. The glass slide with the blood smear on it was then fixed in methanol, followed by methylene blue and eosin staining on an automated fixation Sysmex SP-10 machine at Bristol Royal Infirmary Haematology laboratory. Three blood films per animal were produced and analyzed for any evidence of microcytic anemia by a Consultant Pediatric Haematologist. The remaining blood sample was then run through the Sysmex XN-20 in Bristol Royal Infirmary Haematology department to obtain a differential full blood count, under the instruction of section leader. Serum urea was measured using the Roche Cobas system with reagents and protocols supplied by the manufacturer.

### Histology and electron microscopy

Tissues were fixed in 10% buffered neutral formalin (Merck, Cat# HT501128), further processed and paraffin embedded. 3 μm sections were stained using Periodic acid Schiff (PAS) staining kit (Sigma, Cat# 395B) and Martius Scarlet Blue (MSB) staining kit (Atom Scientific Ltd. Cat# RRSK2-100) according to manufacturer’s instructions). Tissues were imaged using a Leica DM2000 microscope and micrographs taken with Leica Application Suite X software (Leica Microsystems, RRID:SCR_013673). Image analysis was performed using ImageJ (RRID:SCR_003070); all images were contrast enhanced using the same parameters.

Tissues for electron microscopy were fixed in 0.1 M sodium cacodylate, 2% glutaral-dehyde, and imaged on a Technai 12 transmission electron microscope. Average slit diaphragm, foot process and glomerular basement membrane width were calculated using ImageJ (RRID:SCR_003070) assessing at least 20 regions of glomerular basement membrane from at least 3 glomeruli per mouse.

### Immunofluorescence staining of kidney sections

Frozen kidneys were sectioned at 5μm. Sections were blocked in phosphate buffered saline (PBS) containing 3% Bovine Serum Albumin (BSA, Merck Cat# A9647) and 0.3% Triton X-100 for 1 h, then incubated with primary antibodies overnight at 4°C (nephrin 1:300; Gb3 1:100; PECAM 1:50; C3b 1:100, CFH 1:100; C7 1:100; C9 1:100). Following 3 PBS rinses, sections were incubated with fluorophore-conjugated secondary antibodies (Life Technologies) for 1 h at room temperature. For fibrinogen immunofluorescence a FITC-conjugated antibody (Dako, Cat# F0111) was used at a concentration of 1:100 for 1 h at room temperature. Tissues were imaged using a Leica DM2000 microscope and micrographs taken with Leica Application Suite X software (Leica Microsystems, RRID:SCR_013673). Image analysis was performed using ImageJ (RRID:SCR_003070); all images were contrast enhanced using the same parameters.

### RNAscope in situ hybridisation

Tissues were fixed in 4% paraformaldehyde, processed and paraffin embedded. 3 μm sections were then prepared for RNAscope chromogenic single-plex probe analysis. Slides were baked in a dry oven for 1 h at 60°C before being deparaffinised with xylene twice for 5 min, followed by 100% ethanol twice for 1 min. Slides were air dried for 5 min at room temperature. Hydrogen peroxide was applied to each section and target retrieval performed by heating sections for 20 min in target retrieval reagent (Biotechne, Cat# 322000) at 98°C−100°C. Protease plus (Biotechne, Cat# 322330) was applied for 30 min at 40°C. For VEGF-A detection (Biotechne, Cat# 405131) sections were subject to additional amplification steps with AMP1, AMP2, AMP3, AMP4, AMP5, AMP6, AMP 7, AMP 8, AMP 9 and AMP 10 reagents for 30, 15, 30, 15, 30, 15, 15, 30, 60 and 15 min respectively. Before adding each AMP reagent, samples were washed twice with washing buffer (Biotechne, Cat# 310091). The samples were then counterstained with 50% Gill’s haematoxylin I (Sigma, Cat# GHS132) for 2 min at room temperature, rinsed with tap water, followed by another tap water rinse. Samples were dried for 15 min in a 60°C dry oven. Vector Mount mounting media (Vector Laboratories, Cat# H-5000) and cover slips were then added to slides before imaging using a Leica DN2000 microscope and Leica Application Suite software (Leica Microsystems, RRID:SCR_013673).

### Immunofluorescence staining of human cells

Cells were grown in 6 or 12 well plates with glass coverslips placed in each well. Once fully differentiated cell treatments were applied directly or to the transwell compartment above. Once the assay was completed, cells were washed 3 times in PBS and fixed with ice-cold 4% PFA (paraformaldehyde). After 10−15 min PFA was washed off with PBS and permeabilised with 0.3% Triton X-100 for 10 min. All cells were blocked in 3% BSA (Merck, Cat# A9647) for 60 min. Primary antibodies were then applied to the coverslips which were left at 4°C overnight. The following day, cells were washed 3 times in PBS and secondary fluorescent antibodies applied for 1 h at room temperature. Coverslips were then washed again 3 times and mounted with VECTA Shield hard setting mount with DAPI nuclear stain (Vector Lab Inc, Cat# H100). Slides were then imaged using the Leica DMI 6000B microscope or Leica Confocal SP5-II confocal laser scanning microscope at the Wolfson Imaging Institute, University of Bristol; or with the Quorum Spinning Disk 2 confocal laser microscope at the SickKids Imaging Facility, Toronto, Canada.

### Human samples

All studies on human kidney tissue were approved by international and local research ethics committees (REC) and conducted in accordance with the tenets of the Declaration of Helsinki. Renal biopsy samples were obtained from Arkana Laboratories, Little Rock, Arizona, USA (Histopathology Department) and were archived anonymously (REC Protocol #2022/04/9, IRB00008523). The Solutions Institutional Review Board approved this study as minimal risk research as the data collected were those typically obtained for routine clinical practice. Thus, the requirement for informed consent was waived. All samples were processed using standard techniques. Biopsy samples for light microscopy were fixed and transported in neutral buffered formalin. The tissue was dehydrated in a stepwise fashion in graded ethanol solutions. The ethanol was removed with graded xylene solutions, and the tissue was embedded in paraffin. Serial 3μm sections were cut and stained with haematoxylin and eosin or periodic acid−Schiff using standard reagents.

### Immunohistochemistry for C5b-9 in human kidney tissue

Cortical renal tissue was transferred to 4% (wt./vol) paraformaldehyde (PFA) for 48 h. Dehydration and paraffin embedding were performed at by Histology services at Arkana Laboratories, Little Rock, Arizona, USA. Sections (4μm) were cut using a microtome and transferred to glass slides. Deparaffinisation and hydration steps involved incubation in Histoclear II (National Diagnostics, Cat# HS-202) and rehydrated through a graded alcohol series. Antigen retrieval was performed by heating sections in 10mM sodium citrate buffer (pH 6). Sections were quenched using 3% H2O2. Non-specific IgG binding was blocked with 1% BSA and 10% normal goat serum in TBS-Triton-X (0.1%). C5b-9 antibody (Abcam, Cat# ab66768, 1:400) or an IgG control were applied to sections overnight at 4°C. Sections were washed then incubated with SignalStain Boost detection reagent (Cell Signaling Technology, Cat# 8114) for 30 min at room temperature. SignalStain DAB substrate kit (Cell Signaling Technology, Cat# 8059) was applied for 1−2 min, and the sections dehydrated and mounted in DPX (Sigma, Cat# 06522). Tissues were imaged using a Leica DM2000 microscope and micrographs taken with Leica Application Suite X software (Leica Microsystems; RRID:SCR_013673).

### Antibodies

Antibodies used for immunofluorescence were obtained from 2B Scientific (nephrin #BP5030); Beckman Coulter (Gb3 #IM0175); Hycult Biotech (anti-mouse C3b #HM1065 and anti-mouse Factor H #HM119); Abcam (PECAM #ab124432); Quidel (anti-human C3d #A207 and anti-human Factor H #A312); and developed by Professor Paul Morgan (C7 and C9). For detection of complement factor H (CFH) binding by flow cytometry, purified CFH (CSL Behring, Marburg, Germany) that was tagged with Alexa Fluor 488 (concentration 2 mg/mL). For each 100μL of cell suspension, 2μL (4μg) of Alexa Fluor 488-tagged CFH was added.

## Quantification and Statistical Analysis

Statistical analysis was performed using GraphPad Prism 9 (RRID:SCR_002798). When comparing two groups t-tests were used. When comparing more than two groups ANOVA was used with appropriate post hoc analysis. Statistical tests used, and n numbers are shown in figure legends. Data are presented as the mean and error bars represent standard error of the mean. For survival characteristics Kaplan−Meier survival plots were generated, and log rank analysis performed. p values less than 0.05 were deemed statistically significant.

## Supplementary Material

Supplementary Material

## Figures and Tables

**Table 1:** Clinical details of cases whose kidney biopsies were stained with C5b-9

Case ID	Clinical summary	Consultant histopathologist biopsy report	Glomerular C5b-9
Control 1	18-year-old female with urinary protein: creatinine ratio (uPCR) of 87 and normal serum creatinine	healthy appearances	negative
Control 2	8-year-old female with Warburg syndrome and new onset nephrotic range proteinuria; chronic lung disease; healthy renal function	healthy appearances	negative
Control 3	21-year-old male with intermittent frank hematuria; healthy renal function	healthy appearances	negative
STEC HUS 1	43-year-old female with 1 day history of nausea and vomiting; upon admission, found to be anemic and thrombocytopenic with a serum creatinine of 344 μmol/L; treated with PLEX; biopsied 14 days after symptom onset; Shiga-toxin-producing *E. coli* documented	acute glomerular TMA	positive
STEC HUS 2	62-year-old female with 3 days history of bloody diarrhea; thrombocytopenic and anemic; PLEX started but stopped when ADAMTS13 found to be normal; kidney dialysis required and biopsied 15 days after symptom onset; Shiga toxin HUS documented	acute glomerular TMA	positive
STEC HUS 3	2-year-old male with diarrhea and found to have Shiga toxin HUS infection; kidney dialysis required upon admission; biopsied 4 months after presentation	chronic active TMA	positive
VEGF inhibitor 1	67-year-old female with AKI and proteinuria on Avastin; creatinine 468 μmol/L; proteinuria 9 g in 24 h	chronic TMA	positive
VEGF inhibitor 2	65-year-old female with nephrotic range proteinuria on Avastin for metastatic ovarian cancer	acute glomerular TMA	positive
Atypical HUS 1	4-year-old female with microangiopathic anemia and hematuria; identified with alternative complement pathway mutation (factor H mutation)	acute glomerular TMA	positive

## Data Availability

All data reported in this paper will be shared by the lead contact upon request. This paper does not report original code. Any additional information required to re-analyse the data reported in this paper is available from the lead contact upon request.

## Context and Significance

The most common cause of kidney failure in children is due to toxin-producing bacteria that enter the circulation through the gut resulting in a disease called hemolytic uremic syndrome (HUS). This commonly requires kidney dialysis, with approximately 5% of children developing life-long kidney failure or dying. It has been unclear why this toxin has a predilection for the kidney and what its mechanism of action is. This study shows the kidney podocyte cell is an important target of the toxin and signals to blood vessels through cellular crosstalk, causing complement activation and HUS.

When inhibiting complement activation early, HUS is prevented. These results suggest that the podocyte is a key cell targeted in toxin-induced HUS and that early complement inhibition is therapeutically beneficial. to affect multiple organ beds including the kidneys, brain, gastrointestinal tract, respiratory system, and skin.^3^

## Highlights

The podocyte is likely the reason Shiga toxin targets the glomerulus in STEC-HUS

Podocyte-to-glomerular endothelial cell “crosstalk” occurs in STEC-HUS

The complement cascade is activated in STEC-HUS

Early inhibition of the terminal complement pathway ameliorates STEC-HUS
