# Supplementary material for: Shiga toxin targets the podocyte causing hemolytic uremic syndrome through endothelial complement activation
Source: Med. Author manuscript; Available in PMC 2025 Apr 26. (PMC7617617; doi:10.1016/j.medj.2023.09.002)
Supplement: Supplementary Material [file EMS204785-supplement-Supplementary_Material.pdf]

Med, Volume *4*

Supplemental information

Shiga toxin targets the podocyte causing hemolytic uremic syndrome through endothelial complement activation

Emily E. Bowen, Jennifer A. Hurcombe, Fern Barrington, Lindsay S. Keir, Louise K. Farmer, Matthew D. Wherlock, Carolina G. Ortiz-Sandoval, Valentina Bruno, Arlette Bohorquez-Hernandez, Daniel Diatlov, Niyousha Rostam-Shirazi, Sara Wells, Michelle Stewart, Lydia Teboul, Abigail C. Lay, Matthew J. Butler, Robert J.P. Pope, Eva M.S. Larkai, B. Paul Morgan, John Moppett, Simon C. Satchell, Gavin I. Welsh, Patrick D. Walker, Christoph Licht, Moin A. Saleem, and Richard J.M. Coward

Supplementary Information

Shiga toxin targets the podocyte causing Haemolytic Uraemic Syndrome through endothelial complement activation.

Bowen E, Hurcombe J, *et. al.*

List of Supplementary Figures

Figure S1. Generation of PodrtTA-Tet-O-Gb3 Gb3 null (Pod Gb3) mice, related to Figure 1.

Figure S2. Shiga toxin causes Haemolytic Uraemic Syndrome via the podocyte Gb3 receptor- time course, related to Figures 1 and 2.

Figure S3. C5b-9 staining of human renal biopsy cases of STEC HUS and Controls, related to Figure 3.

Figure S4. VEGF-A expression and time-course in GB3 null and Pod GB3 mice, related to Figure 4.

Figure S5. Human Podocyte and GEnC co-culture model set up, related to Figure 5.

Figure S6. Human GEnC monocultures exposed to Stx2 activate complement which is rescued by VEGF-A, related to Figure 5.

Figure S7. Confirmation of C5 inhibition in PodGb3 BB5.1 treated mice and co-localisation of complement deposition in the glomerular endothelium of Stx treated mice, related to Figure 6.

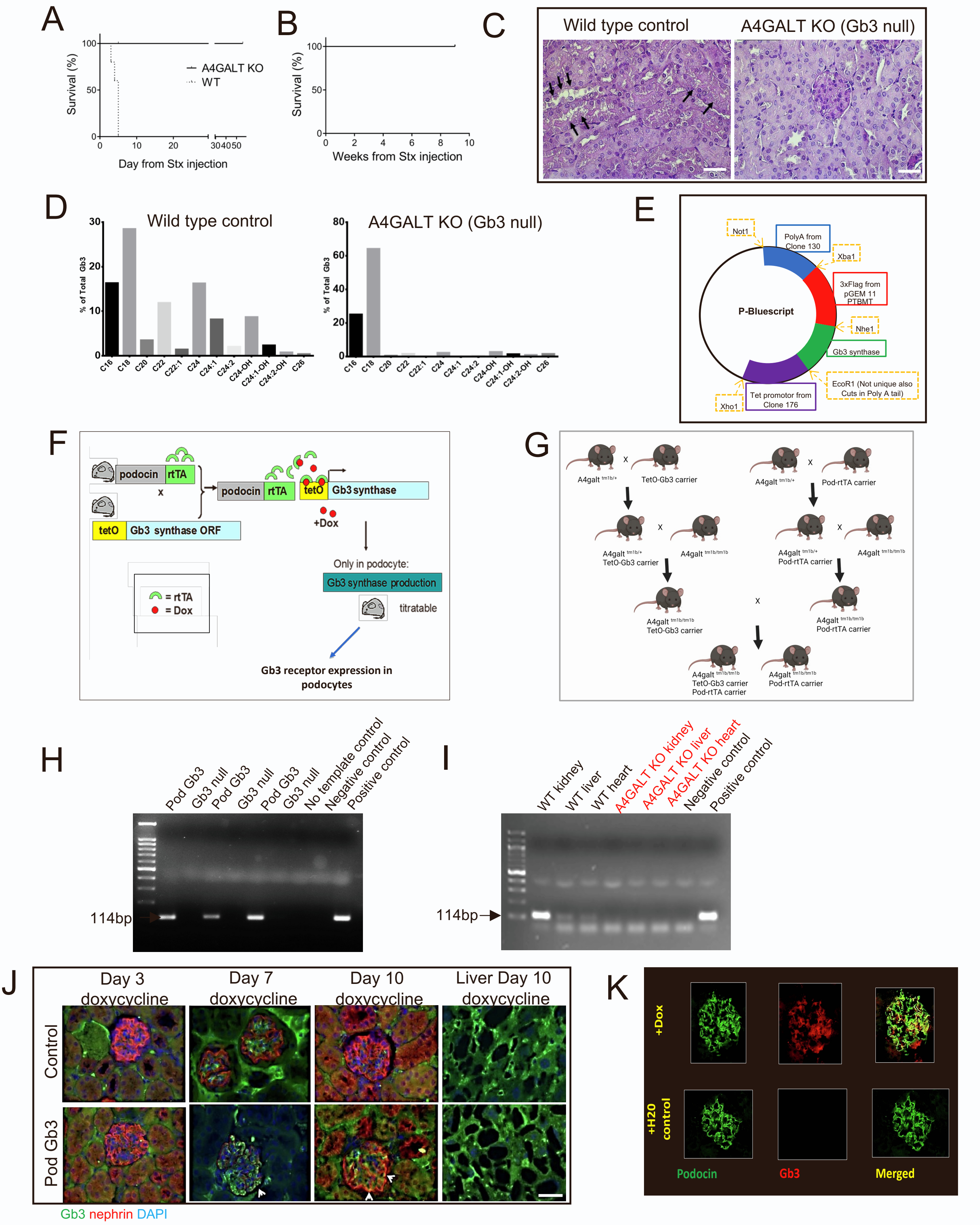

Figure S1. Generation of PodrtTA-Tet-O-Gb3 Gb3 null (Pod Gb3) mice.

A. Kaplan-Meyer plot showing percentage survival of wild type mice (WT n=4) and A4GALT KO (Gb3 null n=4) mice following 10 ng/g intraperitoneal Shiga toxin inoculation.

B. Kaplan-Meyer plot showing percentage survival of A4GALT KO (Gb3 null n=2) mice following 100 ng/g intraperitoneal Shiga toxin inoculation.

C. PAS staining of wild type (WT) mouse kidney tissue following Stx inoculation shows evidence of acute tubular necrosis: oedematous tubules and vacuolations indicated by arrows vs. A4GALT KO (Gb3 null) mouse kidney tissue following Stx inoculation with normal tubular and glomerular morphology. Scale bar, 25 μm.

D. Lipid mass spectrometry analysis for Gb3 isoforms in WT mouse kidney vs. A4GALT KO (Gb3 Null) kidney. Of interest the C24 and C24-1 isoforms of Gb3 are significantly lower in the A4GALT KO mouse. These are known to be the key biologically active isoforms *in vivo*.

E. Tet-O-Gb3 synthase construct used to generate Tet-O-Gb3 mice.

F. Generation of the PodGb3 mouse using inducible tetracycline-controlled transcription of Gb3 synthase (A4GALT) in the podocyte. rtTA=reverse tetracycline-controlled transactivator. Dox=doxycycline.

G. Breeding strategy for the generation of the PodrtTA-Tet-O-Gb3 null mouse on a Gb3 null background: created using Biorender.com.

H. Whole kidney tissue endpoint PCR for Gb3 synthase (A4GALT) at 114 base pairs from PodrtTA-Tet-O-Gb3 Gb3 null (PodGb3) mice and PodrtTA-Tet-O-WT Gb3 null (GB3 null) mice. NTC=no template control (no cDNA), negative control=Gb3 null mouse control, positive control=WT mouse kidney.

I. Mouse kidney, liver and heart tissue were analysed in both WT and A4GALT KO mice for the presence of Gb3 synthase mRNA. Tissues from the A4GALT KO mice (red) show no evidence of Gb3 synthase expression with absence of a detectable band at 114 base pairs (corresponding to Gb3 synthase mRNA product).

J. PodrtTA-Tet-O-Gb3 WT mice were given doxycycline for 3,7 and 10 days alongside WT control mice. Gb3 expression was detected by IF at days 7 and 10 (indicated by arrows). Gb3 (green), nephrin (red) and DAPI nuclear stain (blue). Representative images for n=3 mice for each genotype. Scale bar, 25 μm. Liver positive control included

K. Pod GB3 mice express Gb3 (red) 10 days after Doxycycline administration. The system is not leaky as Pod GB3 mice given water do not express Gb3. Podocin staining also shown (green).

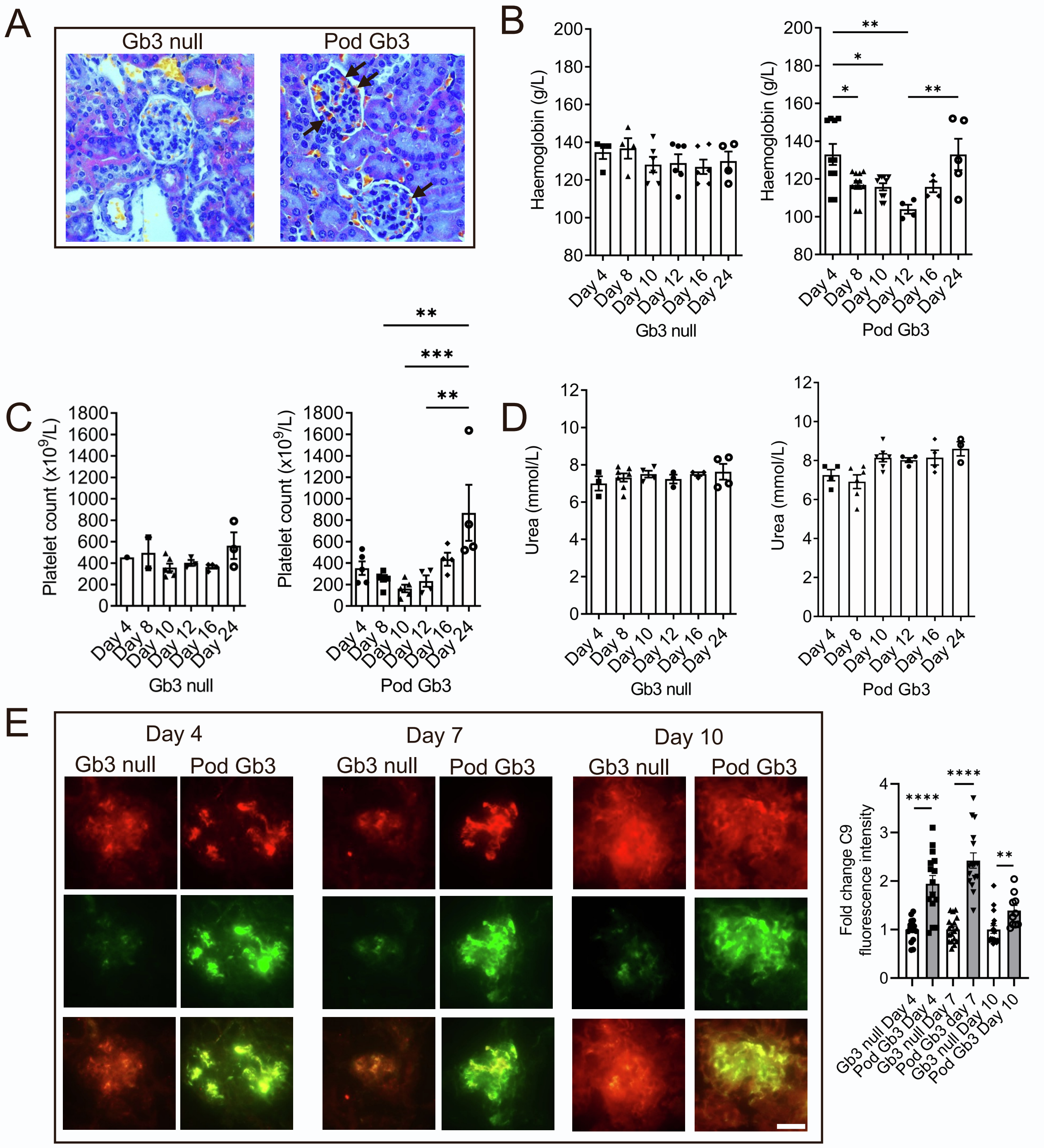

Figure S2. Shiga toxin causes Haemolytic Uraemic Syndrome via the podocyte Gb3 receptor- time course.

A. Martius scarlet blue (MSB) trichrome stain in Gb3 null and pod Gb3 mice at day 10 post-IP Stx. Fibrin = red, collagen = blue, erythrocytes = yellow. Fibrin thrombi indicated by black arrows. Scale bar, 25 μm.

B-D. Blood samples for haemoglobin, platelet count and urea were taken from Pod Gb3 and Gb3 null mice at terminal anaesthesia at timepoints from day 4, 8, 10, 12, 16 and 24 post Shiga toxin intraperitoneal injection. One way ANOVA with Tukey's multiple comparison test: ***p <0.001, **p <0.01, *p<0.05. No significant difference between controls.

E. Glomerular immunofluorescence analysis for complement factor C9 (green) with co-staining for PECAM (red) in GB3 null and Pod Gb3 mice at days 4, 7 and 10 post IP Stx. Scale bar, 25 μm. C9 fluorescence intensity was measured on >10 glomeruli per mouse using Image J . Unpaired t-test ****p<0.0001, **p<0.01.

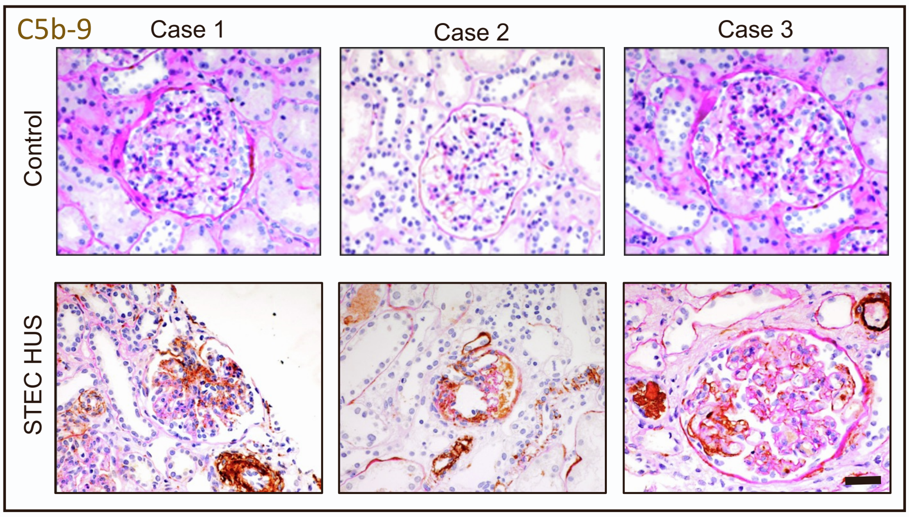

Figure S3. C5b-9 staining of human renal biopsy cases of STEC HUS (cases 1-3) and Controls (cases 1-3).
C5b-9 immunohistochemical staining (brown) shown for 3 cases of STEC HUS vs. 3 cases of control human biopsies. Scale bar, 50 µm.

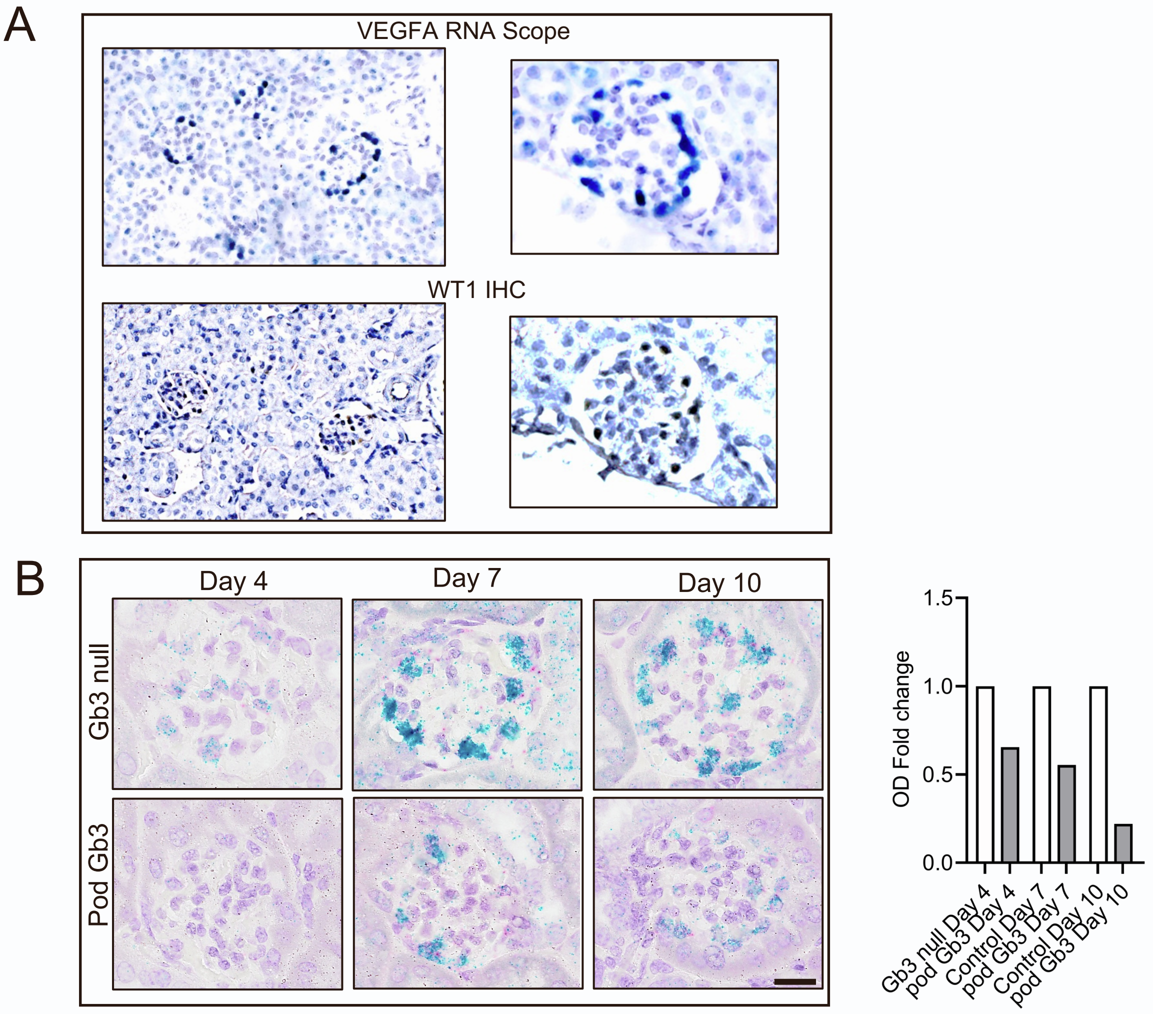

Figure S4. VEGF-A expression and time-course in GB3 null and Pod GB3 mice.
A. Co-localisation of VEGF-A signal with podocyte marker Wilms-tumour 1 (WT1). Serial sections taken and stained for VEGF-A with RNA scope (upper panels) or the use of immunohistochemistry to locate WT1 (lower panels). This shows VEGF-A coming from podocytes.
B. Time course of VEGF-A expression after Stx2 challenge. RNA Scope *in situ* hybridisation shows a reduction of podocyte VEGFA expression in pod Gb3 mice relative to Gb3 null mice at 4, 7 and 10-days post IP Stx. VEGF-A signal intensity was measured in ≥15 glomeruli per mouse using QuPath software. Scale bar, 25 μm.

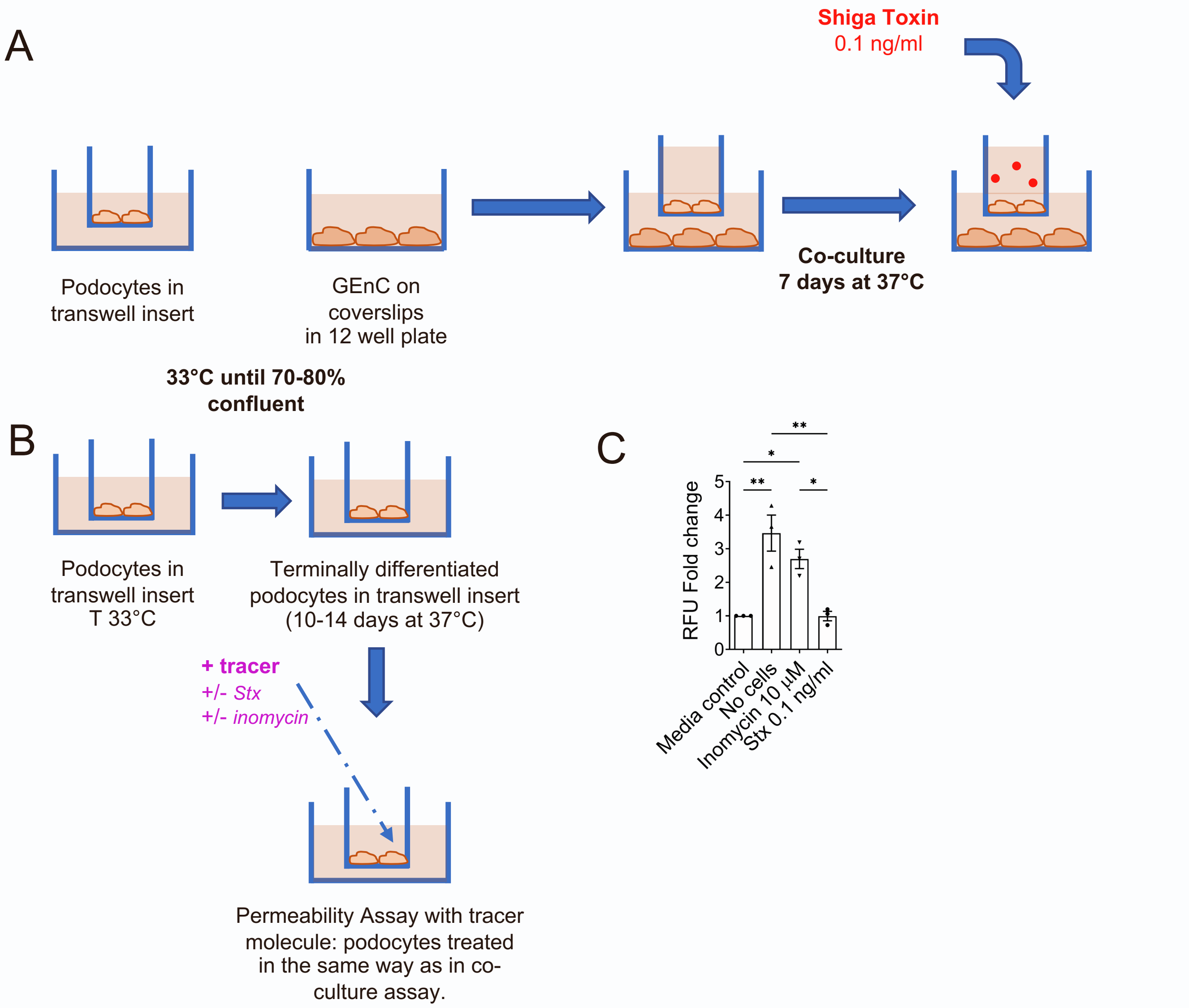

Figure S5. Human Podocyte and GEnC co-culture model set up

A. Co-culture transwell set-up. Podocytes were seeded into the transwell compartment of the co-culture plate and allowed to proliferate at 33°C until the bottom of the transwell was 70-80% confluent at which point they were thermo-switched to 37°C to terminally differentiate. GEnCs were then seeded on to coverslips on a 12 well tissue culture plate and allowed to proliferate until 70-80% confluent at which point the transwell containing differentiated podocytes was transferred to the GEnC 12 well plate and the cells co-cultured for 7 days at 37°C. Podocytes were then treated with Stx.

B. Permeability Assay experiment to assess whether Stx could enter the GEnC compartment of the well. Podocytes were seeded into the transwell compartment of the co-culture plate and allowed to proliferate at 33°C until the bottom of the transwell was 70-80% confluent at which point they were thermo-switched to 37°C to terminally differentiate as before. After 14 days at 37°C a tracer molecule (70kDa dextran – which is the same molecular weight as Stx) + / - Stx, +/- inomycin (used as a way of injuring podocytes to increase their permeability and act as a positive control) were added to the transwell. A transwell with no cells was also used as a positive control for tracer molecule detection. After 15 minutes incubation the plate was read on a bioluminescent plate reader and the light emitted from each well measured.

C. Measured RFU fold change (normalised to media control) shown for each condition. In the Stx treated podocyte transwell there was no detectable dextran tracer molecule indicating the 70kDa tracer molecule does not pass through the transwell. Inomycin was applied to the podocytes as a positive control disrupting the cells and allowing dye to pass through.

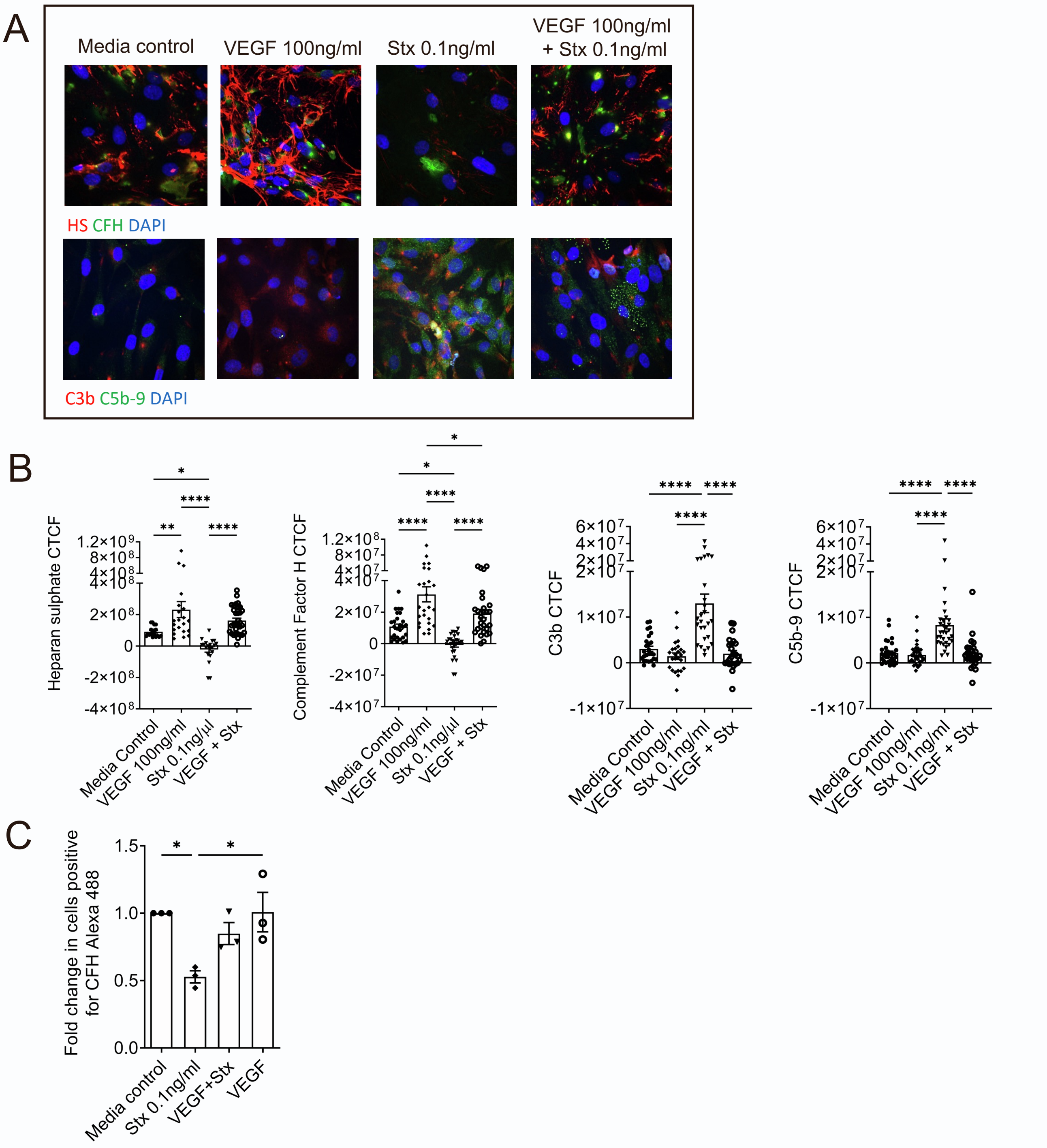

Figure S6. Human GEnC monocultures exposed to Stx2 activate complement which is rescued by VEGF-A.

A GEnC exposed to VEGF-A increase HS and CFH. Shiga-toxin decreases HS, CFH and activates C5-9. This is rescued with extrinsic VEGF-A.

B Quantification of changes described in S5A. One way ANOVA with Tukey's post test, $^*p<0.05$, $^{**}p<0.01$, $^{****}p<0.0001$. Each data point on all graphs represents the average CTCF taken for each field of view, with at least 5 fields of view per condition and n=4 experiments. Data are expressed as mean +/- SEM.

C Complement factor H binding assay in monocultured GENCs analysed for binding of Alexa 488 conjugated complement factor H on flow cytometry. Non-viable cells were excluded from analysis with Fixable Viability Dye eFluor 780. Data normalised to media control and expressed as a fold change in mean fluorescence intensity of Alexa 488 following treatment with Stx 0.1ng/ml, VEGF 100ng/ml + Stx 0.1ng/ml or VEGF 100ng/ml. n=3 experiments with each data point representing an n number. One way ANOVA with Tukey's post-test $^*p<0.05$

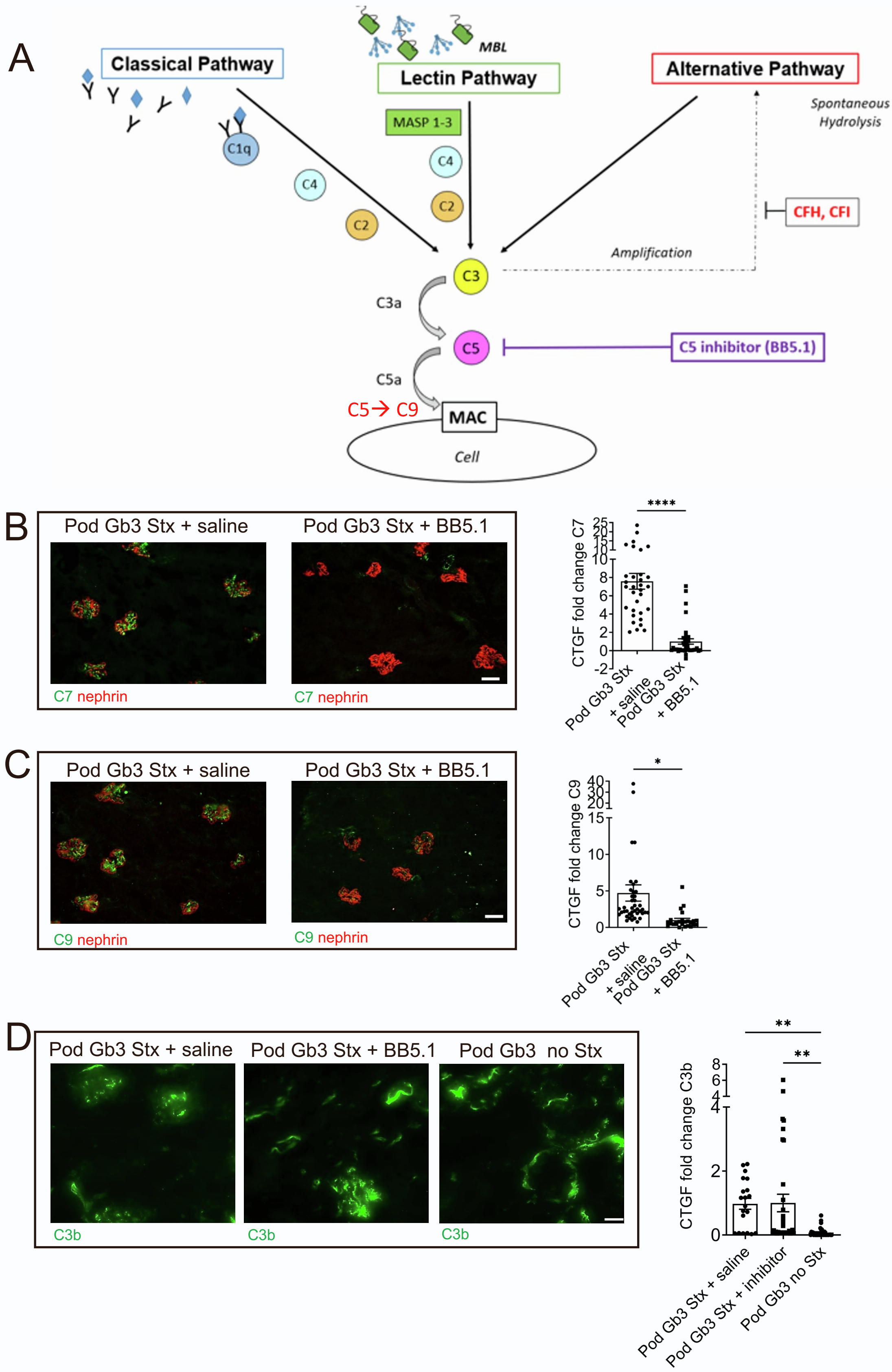

Figure S7. Confirmation of C5 inhibition in PodGb3 BB5.1 treated mice and co-localisation of complement deposition in the glomerular endothelium of Stx treated mice.

A The complement cascade can be activated by one of three pathways: the classical, lectin or alternative pathways. All three converge at the point of C3 cleavage and culminate in the formation of the membrane attack complex (MAC). This leads to insertion of pores in the target cell membrane, osmotic lysis and cell death. C5 inhibition with BB5.1 will not affect C3 activation which occurs upstream of this point in the pathway. However, C5 inhibition will prevent MAC formation and prevent cell death. MBL=mannose binding lectin, CFH=complement factor H, CFI=complement factor I.

B and C Glomerular immunofluorescence analysis for complement factors C7 and C9 (green as indicated) with co-staining for nephrin (red) in PodGb3 mice given Stx and saline control or C5 inhibitor BB5.1. Scale bar, 25 μm. Fold change in corrected total glomerular fluorescence intensity (CTGF) was calculated using Image J analysis for C7 and C9 in the glomerulus. PodGb3+Stx+saline control n=3, PodGb3+Stx+BB5.1 n=3 with 15 glomeruli per mouse analysed. Unpaired T-test ****p<0.0001 and *p<0.05.

D Glomerular immunofluorescence analysis for C3b (green) in Pod Gb3 mice post-IP Stx and either saline or BB5.1. Scale bar, 25 μm. Fold change in corrected total glomerular fluorescence intensity (CTGF) was calculated using Image J analysis for C3b deposition in the glomerulus. Pod Gb3+Stx+saline n=3, Pod Gb3+Stx+Bb5.1 n=3 and Pod Gb3 no Stx untreated control n=3 with 15 glomeruli per mouse analysed. One way ANOVA with Tukey's multiple comparison test **p<0.01.
